# Secondary motoneurons in juvenile and adult zebrafish: Axonal pathfinding errors caused by embryonic nicotine exposure

**DOI:** 10.1002/cne.21903

**Published:** 2009-01-20

**Authors:** Evdokia Menelaou, Kurt R Svoboda

**Affiliations:** Department of Biological Sciences, Louisiana State UniversityBaton Rouge, Louisiana 70803

**Keywords:** neuromuscular junction, fluorescent stereomicroscopy, live imaging, GFP, nicotinic acetylcholine receptor

## Abstract

Nicotine is a drug of abuse that has been reported to have many adverse effects on the developing nervous system. We previously demonstrated that embryonic exposure to nicotine alters axonal pathfinding of spinal secondary motoneurons in zebrafish. We hypothesize that these changes will persist into adulthood. The *Tg*(*isl1:GFP*) line of zebrafish, which expresses green fluorescent protein (GFP) in a subtype of spinal secondary motoneurons, was used to investigate potential long-term consequences of nicotine exposure on motoneuron development. Anatomical characterization of *Tg*(*isl1:GFP*) zebrafish ranging between 3 and 30 days postfertilization (dpf) was initially performed in fixed tissue to characterize axonal trajectories in larval and juvenile fish. *Tg*(*isl1:GFP*) embryos were transiently exposed to 5–30 μM nicotine. They were then rescued from nicotine and raised into later stages of life (3–30 dpf) and fixed for microscopic examination. Morphological analysis revealed that nicotine-induced abnormalities in secondary motoneuron anatomy were still evident in juvenile fish. Live imaging of *Tg*(*isl1:GFP*) zebrafish using fluorescent stereomicroscopy revealed that the nicotine-induced changes in motoneuron axonal pathfinding persisted into adulthood. We detected abnormalities in 37-dpf fish that were transiently exposed to nicotine as embryos. These fish were subsequently imaged over a 7-week period of time until they were ≈3 months of age. These pathfinding errors of spinal secondary motoneuron axons detected at 37 dpf persisted within the same fish until 86 dpf, the latest age analyzed. These findings indicate that exposure to nicotine during embryonic development can have permanent consequences for motoneuron anatomy in zebrafish. J. Comp. Neurol. 512:305–322, 2009. © 2008 Wiley-Liss, Inc.

Exposure of the developing fetus to nicotine through maternal serum has been linked to a number of abnormalities, including spontaneous abortions, low birth weight, and sudden infant death syndrome (DiFranza and Lew,^1995^; Slotkin et al.,^1997^) and causes significant cognitive, intellectual, and behavioral impairments, such as attention deficit hyperactivity disorder (ADHD; Thapar et al.,^2003^; for review, see Slikker et al.,^2005^). Mammalian studies have shown that nicotine-induced abnormalities during critical stages of nervous system development can persist well into adulthood, causing behavioral abnormalities such as increased spontaneous locomotion and impaired fear-associated learning in adult stages (Paz et al.,^2007^). However, the mechanism underlying these abnormalities remains unclear.

In vertebrate central nervous systems (CNS) cholinergic agonists such as acetylcholine and nicotine activate neuronal nicotinic acetylcholine receptors (nAChRs). Acetylcholine-induced nAChR activation has been implicated in regulating pathfinding and the guidance of nerve growth cones in isolated *Xenopus* spinal neurons (Zheng et al.,^1994^). nAChR activation has also been linked to motoneuron survival in the developing chick (Hory-Lee and Frank,^1995^).

In our research, we use the zebrafish model in an effort to define the mechanism by which exposure to nicotine perturbs vertebrate development. Zebrafish are a genetically accessible vertebrate and produce optically translucent embryos, making high-resolution in vivo imaging in embryos and larval fish feasible. Live imaging is a powerful tool for studying axonal regeneration in injured spinal cords in animal models including mice (Kerschensteiner et al.,^2005^), lamprey (Zhang et al.,^2005^), and zebrafish (Bhatt et al.,^2004^). Also, in vivo imaging in transgenic zebrafish expressing green fluorescent protein (GFP) has aided in the visualization and characterization of the vascular (Lawson and Weinstein,^2002^) and lymphatic systems (Yaniv et al.,^2006^). In vivo live imaging offers many advantages over immunohistochemical analysis of fixed tissues, as it eliminates fixation artifacts that could obscure histological features.

The zebrafish motor pool is composed of motoneuron axons that exit ventral spinal cord in each segment and extend their axons to target muscle fibers in the myotome (for review, see Lewis and Eisen,^2003^). We previously reported that chronic nicotine exposure in zebrafish embryos impairs secondary motoneuron axonal pathfinding (Svoboda et al.,^2002^). In that study, anatomical analysis in zebrafish younger than 8 days postfertilization (dpf) was performed using zn5 immunoreactivity to investigate axonal pathfinding. GFP reporter fish were used to examine motoneuron cell biology (i.e., GFP expression) upon nicotine exposure. Since we could reliably detect alterations in motoneuron axonal pathfinding caused by embryonic nicotine exposure, we sought to determine if those alterations persisted into adulthood, or if this anatomical insult recovered as the fish transitioned to adulthood.

To do this, we took advantage of two transgenic GFP reporter fish and characterized their axonal trajectories. In the *Tg*(*isl1:GFP*) zebrafish (Higashijima et al.,^2000^), referred to as *isl1* from here onward, GFP is expressed in a subpopulation of secondary motoneurons innervating the dorsal myotome (Zeller et al.,^2002^). In the *Tg*(*gata2:GFP*) zebrafish (Meng et al.,^1997^), referred to as *gata2* from here onward, GFP is expressed in a subset of secondary motoneurons that innervate the ventral myotome (Zeller et al.,^2002^). The use of the antibody zn5 aided in the characterization of these two transgenic lines early in larval development and helped us identify a suitable reporter fish to examine the long-term consequences of nicotine exposure in juvenile and adult living fish.

Using live imaging techniques, we show that abnormalities in axonal pathfinding resulting from embryonic nicotine exposure persist into adulthood. These findings indicate that exposure to nicotine during embryonic development can have long-term consequences for motoneuron anatomy in zebrafish.

## MATERIALS AND METHODS

### Zebrafish maintenance

Animal protocols were approved by the Institutional Animal Care and Use Committee at Louisiana State University. Fertilized eggs were obtained from natural spawnings of *Tg*(*isl1:GFP*), *Tg*(*gata2:GFP*), and several wildtype lines according to the Zebrafish Book (Westerfield,^1995^). Adult fish were maintained at 28°C with a lighting schedule of 14 hours light and 10 hours dark. Embryos were collected within 3 hours of spawning, rinsed, and placed into 100-mm Petri dishes containing embryo medium. Stage-matched control and nicotine-exposed fish were raised in system water after 5 dpf, cleaned daily (20% water change), and raised on a diet of baby fish food from 5–10 dpf and brine shrimp after 10 dpf. Both experimental and control fish were maintained under the same conditions for the duration of the experiments.

### Nicotine exposures

Zebrafish embryos at 19–22 hours postfertilization (hpf) were exposed while in their chorions to nicotine (5–30 μM, Sigma, St. Louis, MO, Cat. no. N3876-5ml) made in embryo medium (pH 7.2). Nicotine stock solutions were made fresh daily as needed in distilled water and then the stock solution was diluted in embryo medium to obtain final concentrations. Embryos were continuously exposed in 35-mm Petri dishes until 72 hpf. At 48 hpf, all nicotine-exposed and stage-matched control embryos were manually dechorionated. At 72 hpf, nicotine-exposed larvae were transferred into nicotine-free embryo medium and a sample of those larvae (nicotine-exposed and stage-matched controls) were fixed in 4% paraformaldehyde. The remaining nicotine-exposed and stage-matched unexposed (control) larvae were transferred into 1-L beakers containing system water at 5 dpf and raised for examination at later developmental stages.

### Immunohistochemistry

Whole-mount immunohistochemistry was carried out using a modified version of our previously published protocol (Svoboda et al.,^2001^,^2002^; Pineda et al.,^2006^). Briefly, larval zebrafish were first fixed in 4% paraformaldehyde overnight at 2–4°C and then stored in PBST (phosphate-buffered saline [PBS], pH 7.3, containing 0.1% Tween 20). After permeabilization they were incubated in primary antibody overnight at 2–4°C. The monoclonal antibodies zn5 (dilution 1:500) and F59 (dilution 1:250) were obtained from the Developmental Studies Hybridoma Bank (University of Iowa, Iowa City, Iowa) and were used to reveal secondary motoneuron somata and their axons (Fashena and Westerfield,^1999^) and slow muscle fibers (Devoto et al.,^1996^), respectively. The following day the larvae were washed for 90 minutes and then incubated for another 90 minutes in an antimouse fluorescent secondary antibody conjugated to Alexa 546 or Alexa 488 (1:1,000 dilution in PBST; Molecular Probes, Eugene, OR). They were then rinsed in PBST for another 60 minutes and prepared for image analysis. The zn5 mouse monoclonal antibody recognizes a thick band (doublet) at about 75 kD on Western Blots. The zn5 antigen peptide sequences (peptide 1: KHVTGPNQVSTPDTFQIRYPQ; peptide 2: KVSLQVVSQSPITEG) match a stretch of 37 amino acids within the extracellular domain of the zebrafish DM-GRASP sequence (Fashena and Westerfield,^1999^). The zn5 antibody in this study revealed the same stereotypical morphology of secondary motoneurons and their axons as well as the apposed membrane regions of slow muscle fibers (Devoto et al.,^1996^; Fashena and Westerfield,^1999^; Ott et al.,^2001^). The antibody zn5 is currently available as zn8 from the Developmental Studies Hybridoma Bank. The antibodies zn5 and zn8 are duplicate isolates of the same hybridoma (Kawahara et al.,^2002^). The mouse monoclonal antibody F59 is raised against chicken myosin (Crow and Stockdale,^1986^) and this antibody is specific for the fast isotypes of myosin heavy chain in chicken. The specific isotypes of myosin heavy chain recognized by F59 in zebrafish are not known. Staining with this antibody (F59) revealed the distinct labeling pattern of slow muscle fibers in zebrafish (Devoto et al.,^1996^).

### Neuromuscular junction staining

Tetramethylrhodamine conjugated to α-bungarotoxin (Rh-α-btx) (Molecular Probes) was used to identify potential sites of neuromuscular junctions. Transgenic larval zebrafish were processed for immunohistochemistry and then incubated in Rh-α-btx (10 μg/mL) for 90 minutes before image analysis. For double-labeling experiments in wildtype larvae, incubation in Rh-α-btx (10 μg/mL) for 90 minutes was performed first before adding the zn5 antibody to the primary solution (overnight at 2–4°C). The following day the larvae were washed for 90 minutes and then incubated for another 90 minutes in an antimouse fluorescent secondary antibody conjugated to Alexa 488 (1:1,000 dilution) to reveal zn5 labeling.

### Image acquisition and analysis of morphological data in fixed tissue

**Figure 1:**
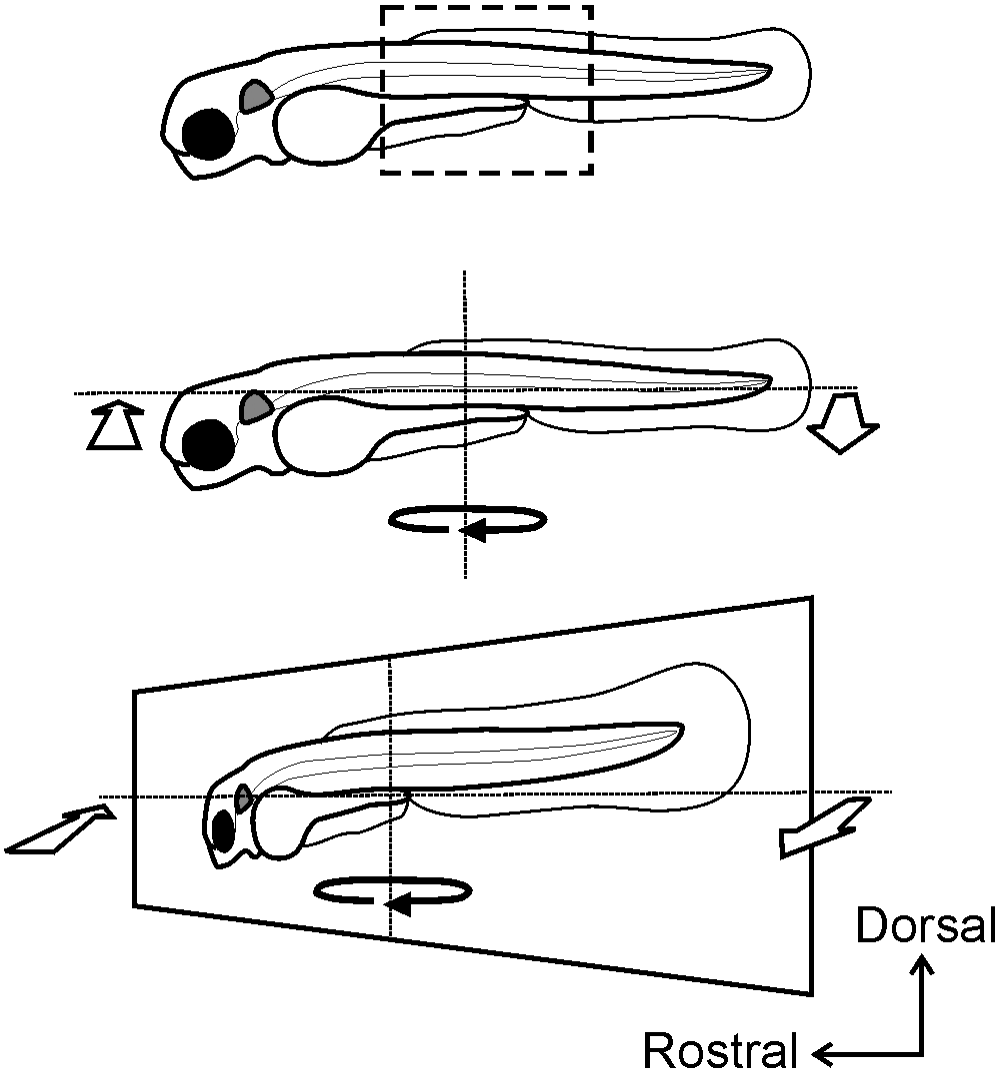
Illustration depicting imaging methodology in fixed tissue. Zebrafish at various ages were analyzed via fluorescent microscopy with the aid of an ApoTome. All zebrafish were mounted on the side. Image stacks were acquired within the region denoted by the dashed box (top) and subsequent examinations using Imaris software included volume rendering allowing rotations in any direction. All rotations shown in this work are presented as horizontal rotations of the dorsoventral axis (middle, black arrow indicates rotational direction) with rostral regions moving away (bottom, left open arrow) and caudal regions moving out of the plane of the page (bottom, right open arrow).

Fixed transgenic zebrafish (3–30 dpf) were mounted laterally on a slide in PBST, lightly coverslipped, and sealed. Images were obtained using a Zeiss (Thornwood, NY) Axiovert 200M inverted microscope equipped with epifluorescence and a Zeiss ApoTome. Images were acquired using a 40× oil immersion objective (N.A. 1.30) and z-stacks (20–60 μm thick) were collected at 0.5–0.8 μm intervals. When imaging α-bungarotoxin (α-btx) signals, the step size interval of the z-stacks was 0.3 μm. The GFP and rhodamine signals were visualized using separate filter cubes. The image stacks were obtained over the yolk sac extension (Fig. 1), including at least one complete side (hemi-segment) of spinal cord, and reconstructed in three-dimensional volumes using Imaris 5.7.2 (Bitplane, St. Paul, MN). Volume rendering analysis included 360° rotations in all directions to reveal motoneuron morphology. All of the rotational views presented in this work were always rotated horizontally around the dorsal–ventral axis with rostral regions moving away and caudal regions moving out from the plane of the page (illustrated in Fig. 1). A total of 268 z-stacks were acquired and analyzed in this study. The model of axonal trajectories in larvae and juvenile *isl1* zebrafish was generated based on observations pertaining to z-stacks obtained from 45 control zebrafish at different developmental stages (3–30 dpf). All images are presented with the rostral region at the left and dorsal to the top as shown in Figure 1. Photoshop 7.0 (Adobe, San Jose, CA) and CorelDraw Graphics Suite 12 (Ottawa, Ontario, CA) were used to crop and organize the figures, respectively.

### Live imaging

*Isl1* zebrafish were first anesthetized in 0.008% tricaine methanesulfonate (MS222) at 17 dpf and in 0.02% MS222 in fish older than 24 dpf. Once completely anesthetized, the fish were placed in a single drop of MS222 on 1-mm-thick slides and imaged live on a Zeiss fluorescent stereomicroscope (Lumar V.12) equipped with an eGFP filter set using a Neolumar S 1.5× objective (150× maximum magnification). Single focal plane images were captured using an AxioCam MRc 5 digital camera. The time interval for live examination was closely monitored. We were able to image anesthetized zebrafish (17 dpf) for 8–10 minutes before they had to be “rescued” and returned back to their individual containers containing system water. In fish older than 2 months of age the imaging interval could be extended to 15–20 minutes. Two or three consecutive live imaging examinations were performed for the same fish given that the fish was allowed to recover from anesthesia for at least 1 hour. This permitted examination of both sides of the fish. During live imaging, fish were kept moist by adding a few drops of MS222. Examination was performed initially at 17 dpf and long-term investigation tracking motoneuron changes in the same fish was carried out every week beginning at 37 dpf and continued until 86 dpf (≈3 months old). At the end of the long-term investigation, all zebrafish were transferred back to their own individual tanks in our zebrafish facility. All acquired images during live examination were digitally processed with the aid of Adobe Photoshop 7.0. The invert function was used to convert the GFP signals to a black-and-white image.

### Statistics

All values are reported as means ± standard error of the mean (SEM). The Mann–Whitney *U*-test was performed to test for significance, which was assigned if the *P*-value was <0.05.

## RESULTS

### Zebrafish secondary motoneuron axons

There are different developmentally regulated populations of motoneurons in the zebrafish spinal cord. Secondary motoneurons have a distinct morphology, physiology, and timing of their development (Myers et al.,^1986^; Westerfield et al.,^1986^). They are born during later stages of development following the primary motoneurons and start extending their axons around 22–23 hpf (Pike et al.,^1992^; Beattie et al.,^1997^). Secondary motoneuron somata are numerous and are located within ventral spinal cord (Myers,^1985^). Their axons exit each segmental root as a nerve bundle following the path established by primary motoneuron axons. They extend into the myotome to contact either medially located (fast, white) or laterally located (slow, red) myotomal muscle fibers (Fetcho,^1986^; Westerfield et al.,^1986^; Pike et al.,^1992^; Devoto et al.,^1996^).

In our previous work, secondary motoneuron development and axonal pathfinding was examined using the *isl1* GFP reporter fish and the zn5 antibody which recognizes the adhesion molecule DM-GRASP (Burns et al.,^1991^). However, the use of zn5 is limited in younger zebrafish (younger than 8 dpf; Svoboda et al.,^2002^) and it can only be used in fixed tissue. Our goal of determining whether nicotine-induced abnormalities in secondary motoneuron axons persisted in fish older than 8 dpf required that we identify other markers of secondary motoneuron axons which could be imaged in living fish.

Transgenic lines of zebrafish provide a great opportunity to visualize GFP expression within axonal trajectories in older living fish. In the *isl1* zebrafish line, GFP expression is driven under direction of the *islet1* promoter. In *isl1* zebrafish older than 54 hpf, GFP can be detected in secondary motoneurons (Higashijima et al.,^2000^). These motoneurons can be identified according to their ventral positions in spinal cord and their axons which travel in nerve bundles projecting into the dorsal musculature (Ott et al.,^2001^; Svoboda et al.,^2002^; Zeller et al.,^2002^). In the *gata2* transgenic line, GFP is expressed in a subpopulation of ventrally projecting secondary motoneuron axons (Meng et al.,^1997^; Pineda et al.,^2006^).

**Figure 2:**
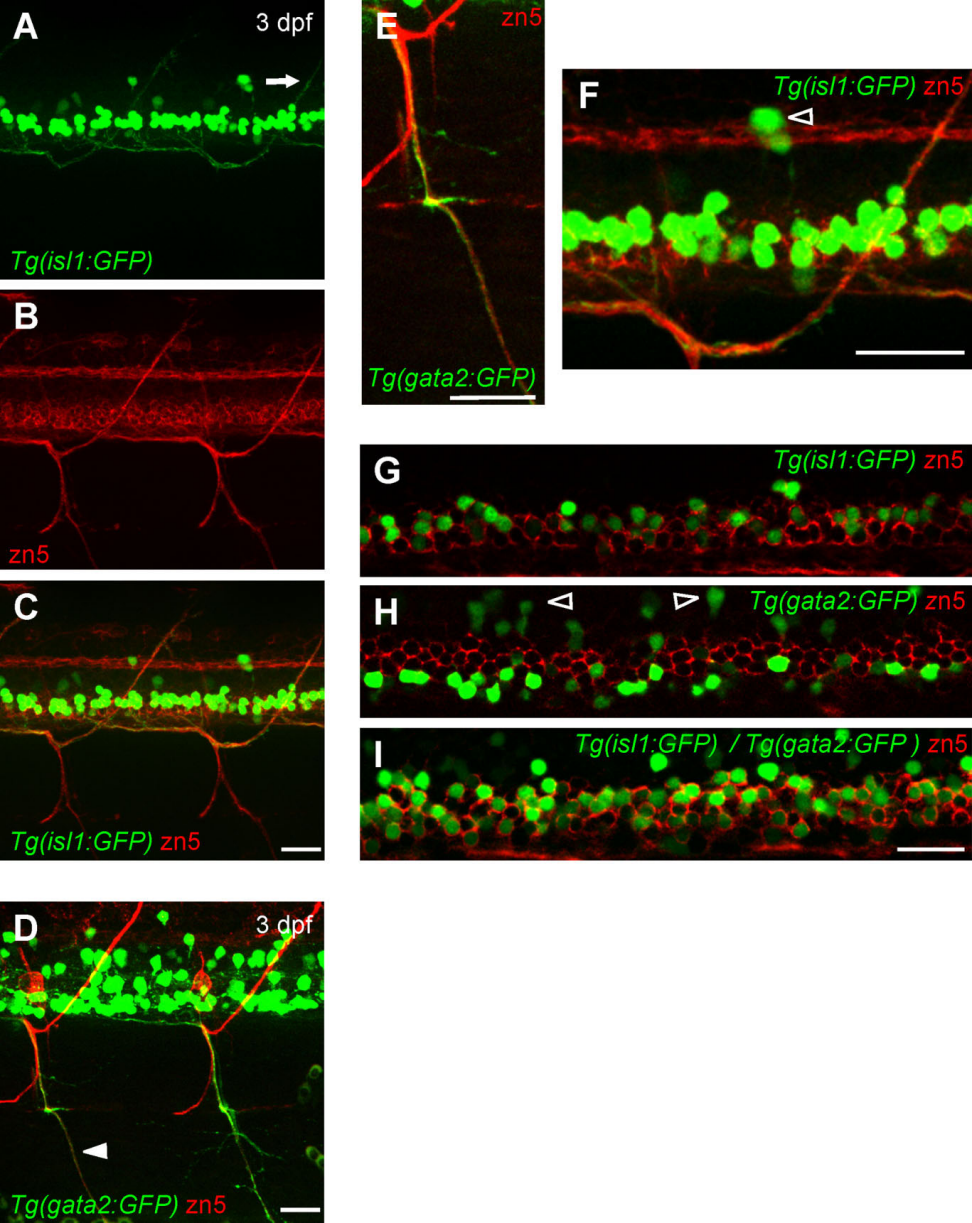
Zn5 labeling of secondary motoneuron somata and their axons in *isl1* and *gata2* zebrafish. **A**: Photomicrograph of a projected z-stack from a 72 hpf *isl1* zebrafish reveals GFP-positive motoneuron somata located in ventral spinal cord. Secondary motoneurons extend their axons dorsally to innervate dorsal musculature (filled arrow). **B**: Zn5-positive axons extend both ventrally and dorsally into the myotome. **C**: The merged image reveals that dorsally projecting GFP-positive axons are recognized by zn5. The GFP signal in spinal cord was saturated to reveal motoneuron axons. **D**: Projected z-stack from a 72-hpf *gata2* zebrafish reveals GFP-positive motoneuron somata and ventrally projecting axons (filled arrowhead) and zn5-positive axons extending both ventrally and dorsally into the myotome. The GFP signal in spinal cord was saturated to reveal motoneuron axons. **E**,**F**: Expanded views from D and C merged images, respectively, show zn5 labeling of ventrally projecting GFP-positive motoneuron axons in a *gata2* larva (left) and on dorsally projecting GFP-positive motoneuron axons in an *isl1* larva (right). GFP-positive cells located more dorsally are presumably interneurons (open arrowhead). **G**–**I**: Single focal plane images selected from a series of stacks from *isl1*, *gata2*, and *isl1*/*gata2* zebrafish indicating that zn5 labels a population of motoneurons other than just the GFP-positive motoneurons in *isl1* or *gata2* zebrafish. Motoneuron somata that express GFP driven by the *islet1* promoter (G) are detected by zn5 and are positioned in mid-dorsal spinal cord. Only a subpopulation of zn5-positive motoneuron somata, which are located in ventral spinal cord, is GFP-positive in *gata2* zebrafish (H). Some interneurons located dorsally are not zn5-positive (open arrowheads). Zn5 immunoreactivity in *isl1*/*gata2* transgenic zebrafish (I) detects most GFP-positive motoneurons. The presence of zn5-positive/GFP-negative cells indicates the presence of other secondary motoneuron subpopulations. A total of 21 fish were analyzed for this figure. A magenta/green copy of this figure is available as Supplemental Figure 3. Scale bars = 20 μm. A–C share the scale bar in C and G–I share the scale bar in I.

Zn5 immunoreactivity exhibits stereotypical labeling of secondary motoneuron somata and their axons that extend within the ventral and dorsal myotome (Fig. 2B). When analyzed in 3 dpf *isl1* zebrafish, dorsally projecting GFP-positive motoneuron axons were zn5-positive (Fig. 2A–C,F). Similar examination in *gata2* zebrafish revealed that zn5 labeled the main GFP-positive ventrally projecting nerve (Fig. 2D,E). When examining zn5 immunoreactivity within spinal cord we noticed that subsets of zn5-positive cells corresponded to GFP-positive somata in *isl1* zebrafish (Fig. 2G) as well as in *gata2* zebrafish (Fig. 2H). In the *isl1* line, GFP/zn5-positive cells lie within mid-dorsal spinal cord (Fig. 2G). In the *gata2 line*, GFP/zn5-positive cells were confined to more ventral spinal cord (Fig. 2H). In *gata2* zebrafish, the dorsally located GFP-positive cells that are zn5-negative are most likely interneurons (Fig. 2H, arrowheads; Hale et al.,^2001^). Mating crosses of *isl1* and *gata2* transgenic GFP reporter fish resulted in transgenic zebrafish expressing GFP in both subpopulations of motoneurons. GFP expression associated with both populations of motoneurons was correlated with most of the zn5-positive cells (Fig. 2I). However, it appeared that not all zn5-positive cells were expressing GFP driven by either the *islet1* or *gata2* promoters. This suggests that there is yet another subclass of secondary motoneurons, which is zn5-positive.

**Figure 3:**
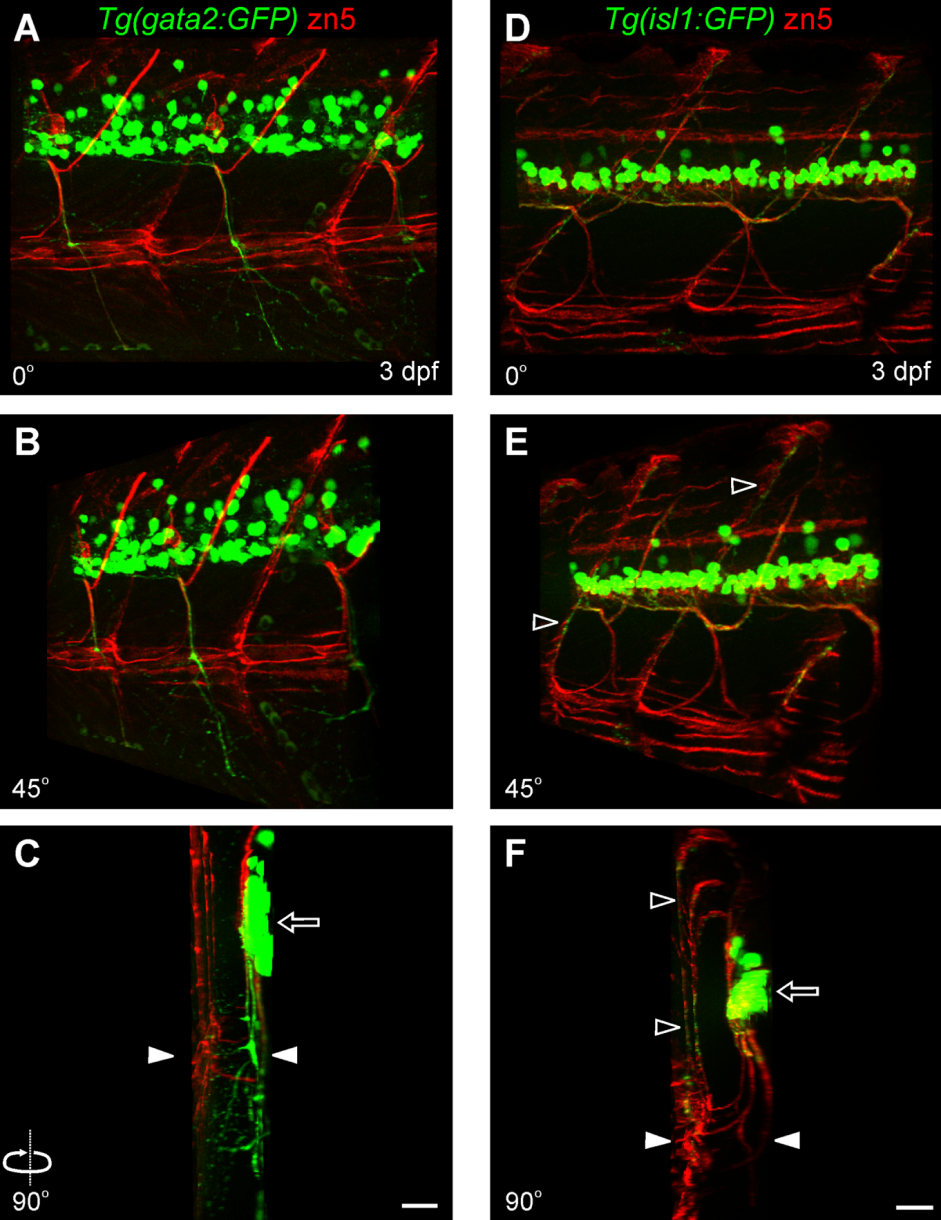
Secondary motoneuron axons in *isl1* and *gata2* zebrafish. **A**: Lateral view of a maximum projection from a z-stack (0° rotation) from a 72-hpf *gata2* zebrafish labeled with zn5 reveals ventrally projecting motoneuron nerves exhibiting secondary and tertiary branches. **B**: Three-dimensional rendering at a 45° rotation reveals axons exiting ventral spinal cord that exhibit branching extending laterally within the myotome. **C**: At a 90° rotation the spinal cord (half spinal hemisegment) is to the right (open arrow) with the most superficial (lateral, zn5-positive) side of the fish to the left. Note the extensive branching from the ventral main nerve within the myotome (GFP signal below the white arrowheads). **D**: Lateral view (0° rotation) from a z-stack from a 72-hpf *isl1* zebrafish reveals zn5 immunoreactivity in dorsally and ventrally projecting axons in addition to the slow muscle boundaries. **E**: Three-dimensional rendering at a 45° rotation reveals zn5-positive axons that project to reach the lateral edge and then take a dorsal path along the segmental boundaries at the most lateral part of the fish (open arrowheads). **F**: The 90° rotation reveals the cross-sectional view showing the left side of the spinal cord to the right (open arrow) with the superficial/lateral side of the fish to the left (open arrowheads). Both zn5- and GFP-positive axons are evidently extending from a medial plane (ventral of spinal cord) out into the most lateral surface of the fish (open arrowheads in E,F). Note the GFP-positive axonal trajectories located within the dorsal myotome (region above the white arrowheads). A total of 21 fish were analyzed for this figure. White arrowheads indicate the midline in C and F. A magenta/green copy of this figure is available as Supplemental Figure 4. Scale bars = 20 μm. A–C share the scale bar in C and D–F share the scale bar in F.

The zn5 antibody also detects the most lateral (superficial) region of the zebrafish where it labels the boundaries of slow muscle fibers (Fashena and Westerfield,^1999^). These slow muscle fibers are superficially located just beneath the skin (Devoto et al.,^1996^). Volume rendering analysis in *gata2* zebrafish (Fig. 3A–C) indicated that the GFP-positive ventrally projecting main nerves had numerous branches extending from them. These axonal branches did not extend to the most superficial layer of the zebrafish, which was revealed by zn5 immunoreactivity, when rotated 90° (Fig. 3C and data not shown). The axons were located within the region where fast muscle fibers make up the myotome and were juxtaposed to Rh-α-btx-positive nicotinic acetylcholine receptors (see Suppl. Fig. 1).

**Figure 4:**
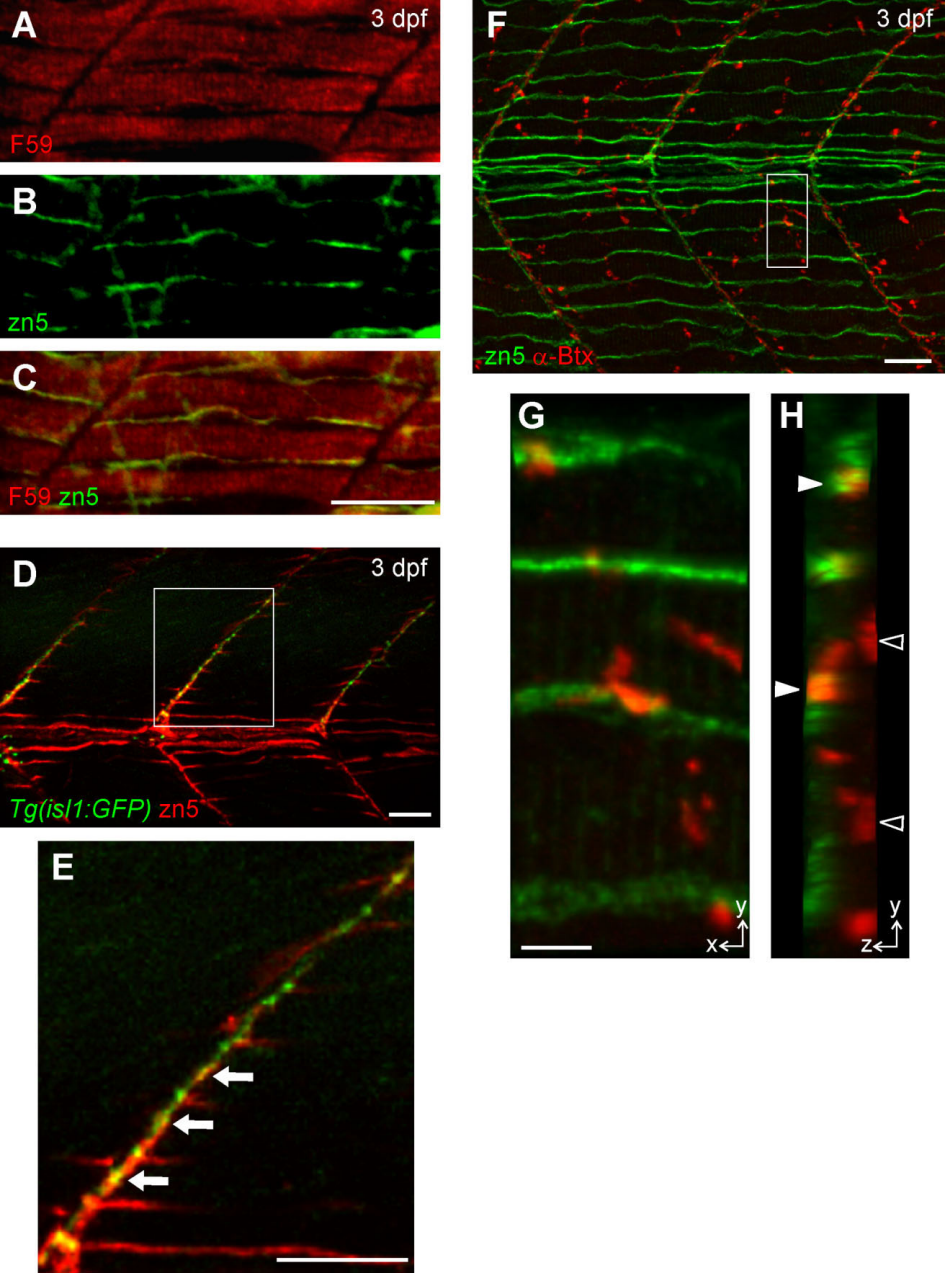
Axonal trajectories in *isl1* zebrafish are associated with laterally located muscle fibers. **A**: Photomicrograph of 72-hpf wildtype larval zebrafish labeled with the antibody F59 to detect slow muscle fibers located at the most superficial part of the fish under the skin. **B**: Zn5 immunoreactivity localizes on superficial muscle cells only on their surfaces apposed to other slow muscle cells. **C**: The merged image reveals double antibody staining with F59/zn5. **D**: In 72-hpf *isl1* zebrafish, GFP-positive secondary motoneuron axons extend into the dorsolateral myotome as shown by localization of the zn5 signals with GFP-positive axons. **E**: A magnified view of the boxed area shown in D demonstrates close association of the lateral most GFP-positive axons with zn5 signals (white arrows). **F**: Rh-α-btx labeling reveals muscle nicotinic acetylcholine receptors at the lateral region of the fish along zn5-positive segmental boundaries. **G**: Magnified lateral view of the boxed area shown in F shows the distribution of muscle nicotinic acetylcholine receptors at the lateral myotome. **H**: Side view (90° rotation) of the area shown in G reveals the spatial distribution of the muscle nicotinic acetylcholine receptors both at the slow (filled arrowheads) and fast muscle (open arrowheads) levels. Superficial/lateral side is to the left and medial is to the right. A total of 16 fish were analyzed for this figure. A magenta/green copy of this figure is available as Supplemental Figure 5. Scale bars = 25 μm in A–C; 20 μm in D–F; 5 μm in G. A–C share the scale bar in C.

Similar analysis was performed in *isl1* zebrafish and revealed GFP-positive motoneuron axons extending dorsally to innervate medially and laterally located musculature (Fig. 3D–F). The zn5 labeling at the segmental boundaries in the lateral periphery were in the same focal plane as superficially located slow muscle fibers (Fig. 4A–C). The lateral axonal trajectory in *isl1* larvae appeared to contact zn5-positive structures at these segmental boundaries (Fig. 4D,E). Neuromuscular junction staining revealed the presence of nicotinic acetylcholine receptors on zn5-positive segmental boundaries and muscle fibers located laterally within the myotome (Fig. 4F–H). Rh-α-btx labeling of the V-shaped segmental boundaries (myosepta) does not necessarily represent synapses (Chen et al.,^1990^; Ono et al.,^2004^). The spatial distribution of the *isl1* axonal trajectories in the lateral myotome suggests that different subpopulations of axons within the main nerve bundle target either fast or slow muscle fibers (see Suppl. Fig. 1). Zn5 labeling thus served as a reference marker of the lateral periphery of the zebrafish myotome and guided us in distinguishing between medially and laterally located motoneuron axons.

The zn5 antibody helped us characterize and identify a suitable transgenic reporter fish for live imaging experiments. The *isl1* line was better suited for our experimental endeavors for the following reasons. First, our previous work showed that zn5-positive dorsal axons of nicotine-exposed embryos exhibited pathfinding errors. Since the zn5 antibody labeled dorsally projecting GFP-positive axons in *isl1* fish, we hypothesized that the nicotine-induced abnormalities detected by zn5 immunohistochemistry would also be present in GFP-positive dorsal axons in *isl1* zebrafish at later stages of development. Second, the *isl1* zebrafish line expresses GFP in more secondary motoneurons (dorsomedial, dorsolateral) but with less axonal arborization (in fixed tissue analysis) early in development. This minimal axonal branching would facilitate the detection and monitoring of nicotine-induced changes. Third, the axonal trajectories in the *gata2* fish exhibited extensive arborization early in development, making it potentially difficult to detect and monitor any nicotine-induced changes, especially if those changes were subtle in nature.

### *Isl1* zebrafish: a working anatomical model of axonal trajectories

**Figure 5:**
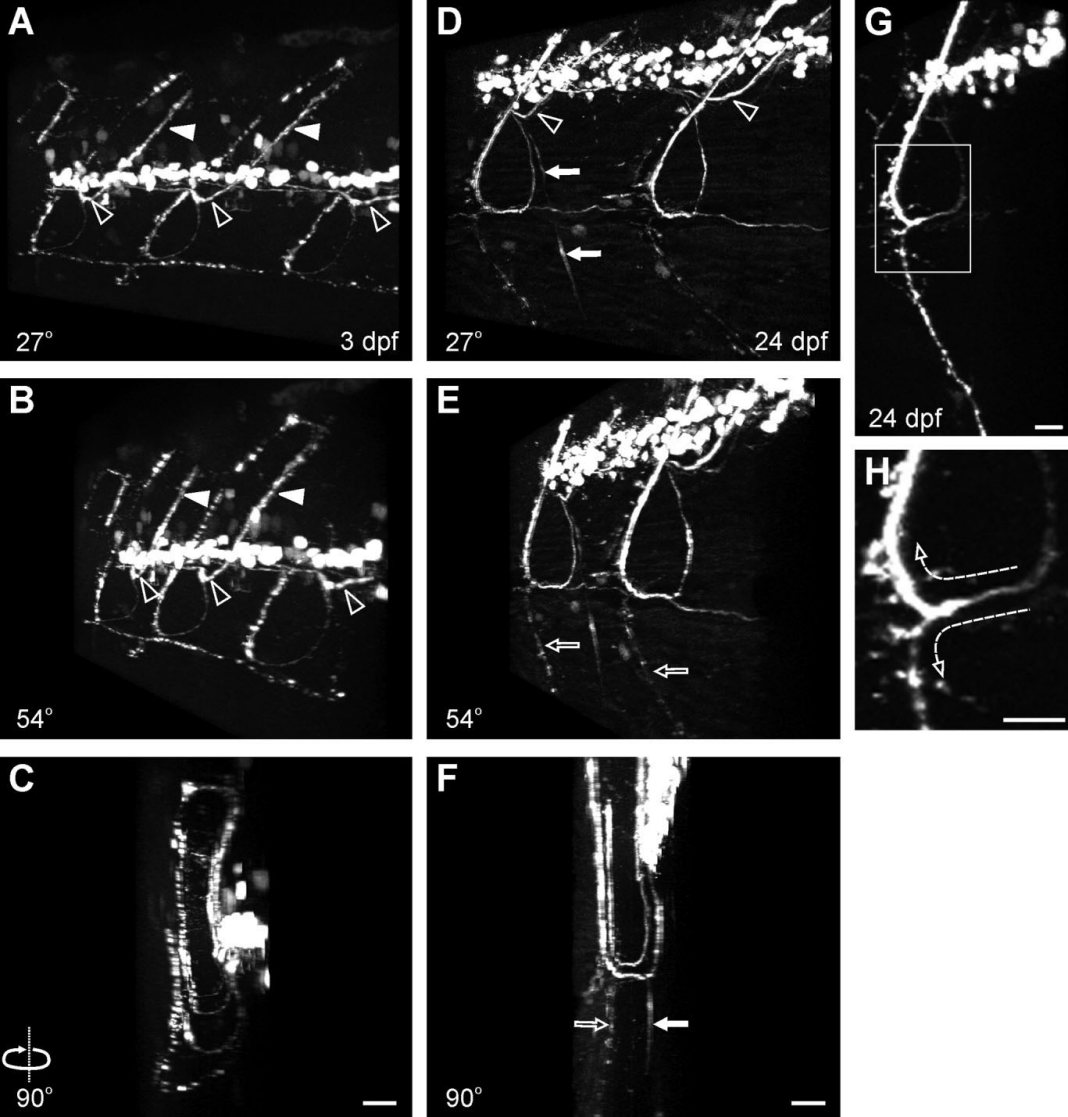
Anatomical characterization of secondary motoneuron axons in *isl1* zebrafish. The developmental progression of secondary motoneuron axons was examined and four distinct secondary motoneuron axon subpopulations were classified based on their anatomical position. **A**–**F**: Projected images from a 3-dpf (A–C) and a 24-dpf *isl1* zebrafish (D–F) are shown at 27° (A,D), 54° (B,E), and 90° (C,F) rotations. One population of axons exits ventral spinal cord from mid-segmental roots and then takes a dorsal turn (“check”-like) (open arrowheads) to extend and innervate musculature within dorsomedial myotome (filled arrowheads). A second population of axons (“loop”-like trajectory) projects ventrally to the midline, then it takes a lateral turn to reach the most lateral periphery and finally project dorsolaterally. A third class of axons exits from ventral spinal cord and continues to project ventromedially passing the midline to innervate the ventral-most musculature of the fish (filled arrows, D,F). At later stages in development, a fourth spatially distinct axonal trajectory is present, extending ventrally at the most lateral part of the fish (open arrows, E,F). **G**: A projected image (49° rotation) of a 24-dpf zebrafish reveals that the axons extending dorsoventrally at the most lateral region of the fish are originating from the same nerve (“loop”-like). **H**: Magnified view of the boxed area in G reveals the divergence of the two axonal trajectories (dashed arrows), each taking their own path innervating either dorsolateral or ventrolateral musculature. Scale bars = 20 μm. A–C share the scale bar in C and D–F share the scale bar in F.

*Isl1* zebrafish were morphologically characterized in fixed tissue at various developmental stages (3, 5, 8, 10, 12, 15, 17, 24, 30 dpf). Optical sectioning was performed to obtain images starting from the most lateral surface of the fish and ending within mid spinal cord. This ensured that entire axonal trajectories would be analyzed as they exit spinal cord and extend into the periphery. Volume rendering of z-stacks helped us identify secondary motoneuron axons as they extended into the myotome at different developmental ages (Fig. 5 and data not shown).

Early in development (3 dpf), motoneuron axons in *isl1* zebrafish have two main distinct trajectories (Fig. 5A–C). First, a bundle of axons exits from each mid-segmental root and takes a dorsal path, with a characteristic “check” pattern (Fig. 5A,B, open arrowheads) extending within the dorsomedial myotome (Fig. 5A,B, filled arrowheads). The second class of axonal trajectories exits at the mid-segmental root and initially extends ventrally and medially. Once these axons reach the horizontal myoseptum, they take a lateral path. When they reach the lateral region of the myotome they then project dorsally. These axonal trajectories have a characteristic “loop”-like pattern (Fig. 5A–C). When rotated 90°, their three-dimensional nature was evident (Fig. 5C).

By 5 dpf a third class of GFP-positive axonal trajectories was evident. These trajectories exit at mid-segmental roots to extend within the ventromedial myotome. These trajectories were also evident at 24 dpf (Fig. 5D,F, filled arrows). A fourth population of GFP-positive axons was also evident by 5 dpf (data not shown) and was easily visualized in the most lateral region of the fish. There, these axons take a ventral path to target muscle fibers located within the ventrolateral myotome. These axons were clearly visualized in 24-dpf juvenile zebrafish (Fig. 5E,F, open arrows). Closer examination revealed that these ventrolateral trajectories originated from the same population of axons that make up the “loop”-like trajectories (Fig. 5G). Evidently, these axons extend laterally along the horizontal myoseptum and reach the lateral region, where they diverge either dorsally or ventrally (Fig. 5H). This lateral region may act as an important guidance choice point where secondary motoneuron axons commit to either the dorsal or ventral path as they project towards lateral muscle fibers.

**Figure 6:**
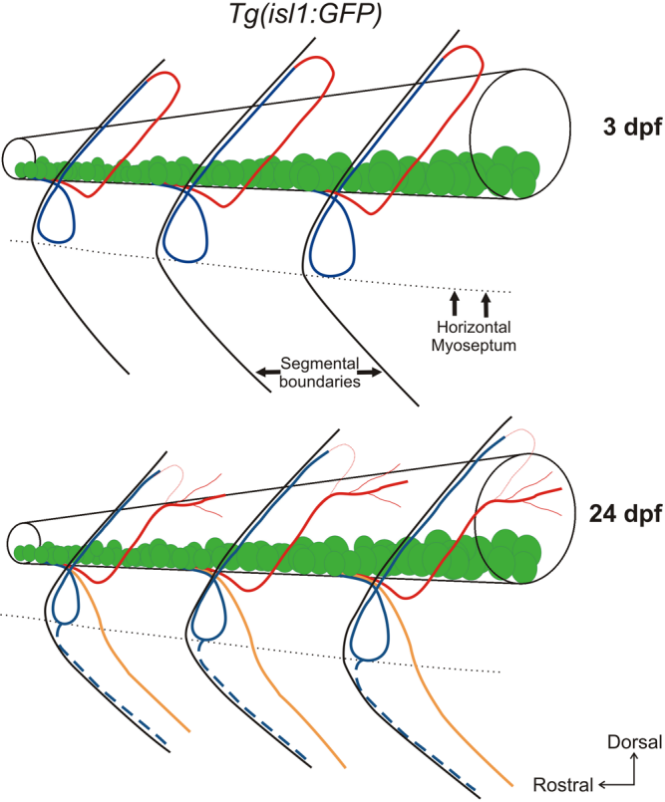
Diagrammatic illustration of the motoneuron axons in *isl1* zebrafish. Cartoons depict the patterning of the secondary motoneuron axons in the *isl1* zebrafish according to our characterization shown in Figure 5. GFP-positive motoneuron somata located in ventral spinal cord are shown in green. Dotted line indicates the horizontal myoseptum. Continuous black lines (V-shaped) represent segmental boundaries. At 3 dpf, a subset of motoneuron somata extend their axons to exit from mid-segmental roots, where they travel shortly in a ventral path and then take a dorsomedial turn (red). Another population of secondary motoneurons axons (shown in blue) exits the same mid-segmental root to follow a ventral path until they reach the horizontal myoseptum. There they turn laterally and finally take a dorsolateral path. At 24 dpf, the motoneuron axons shown in red and blue at 3 dpf maintain their trajectories with the exception that the nerve bundles extending dorsomedially (red) at 24 dpf are putting out secondary and tertiary branches in the dorsomedial myotome. At later stages of development, another nerve is located ventrally and retains a ventromedial trajectory (orange). The motoneuron axons, shown in dashed blue, are diverging from the motoneuron axons shown in blue (dorsolateral) and they take a ventrolateral path. A total of 45 fish between 3–30 dpf were analyzed in generating this model. Cartoons are not drawn to scale. A magenta/green copy of this figure is available as Supplemental Figure 6.

This anatomical characterization revealed four distinct subpopulations of secondary motoneuron axonal trajectories that were classified based on anatomical position. They are schematically illustrated in Figure 6. Axons of GFP-positive motoneuron somata located in ventral spinal cord (Fig. 6) exit from each ventral root in each segment and extend into the myotome. At 3 dpf the first detectable axonal trajectories were seen projecting into the dorsal myotome (Fig. 6, red and blue axons). At later ages of development the main nerve projecting dorsomedially exhibited secondary and tertiary branches (Fig. 6, red axons). Also, additional ventrally projecting axonal trajectories were evident medially and laterally (Fig. 6, orange and dashed blue axons, respectively).

### Nicotine-induced axonal pathfinding errors in *Tg*(*isl1:GFP*) larval and juvenile zebrafish

**Figure 7:**
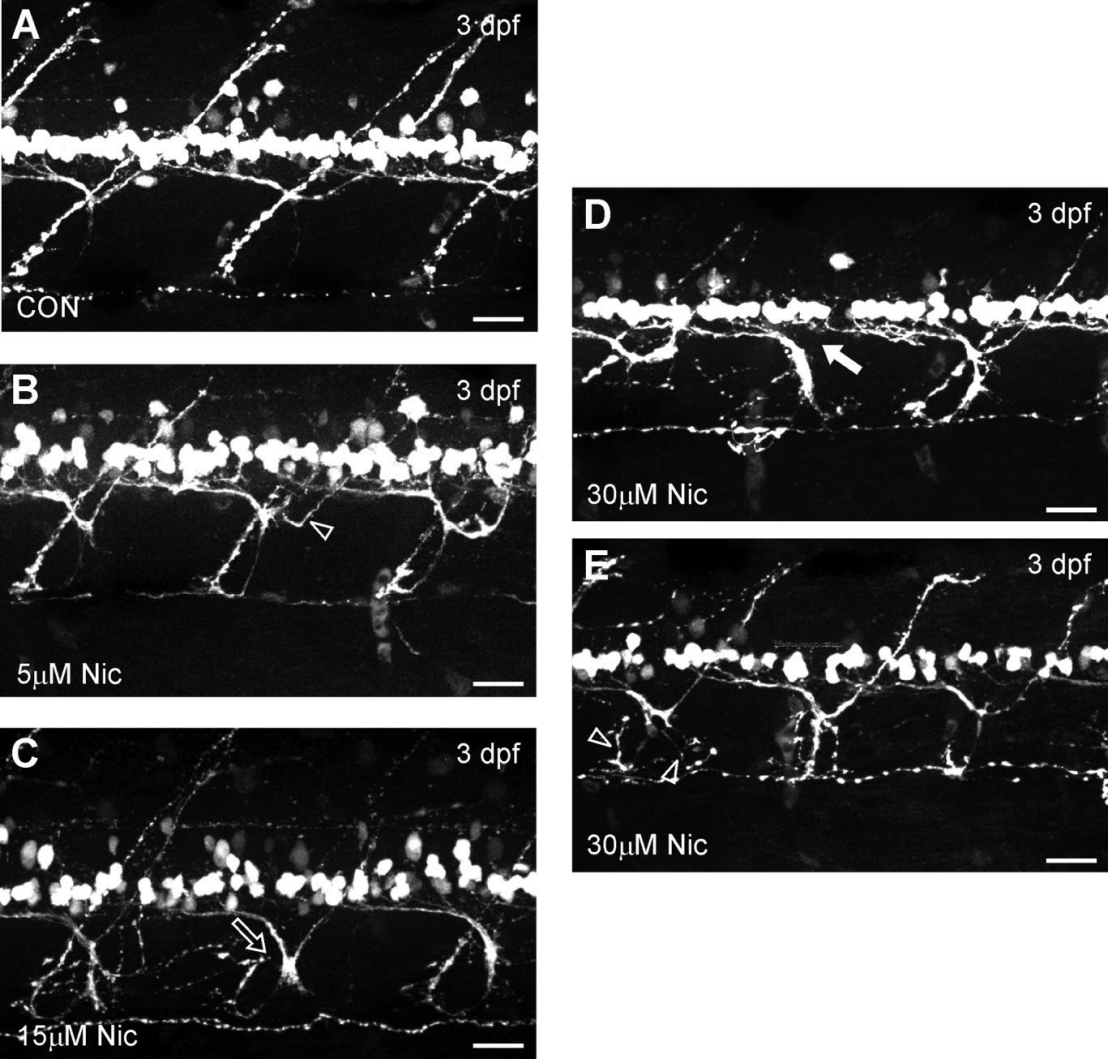
Embryonic nicotine exposure causes secondary motoneuron axonal pathfinding errors early in development. **A**: Projected image from a z-stack shows a stage-matched control *isl1* larval zebrafish (n = 15 fish) that has the characteristic pattern of the “check”-like trajectories and the ventrally projecting axons that loop laterally to take a dorsolateral path in each segment. **B**: *Isl1* zebrafish embryos exposed to 5 μM nicotine (n = 5 fish) have abnormal trajectories exhibiting duplicated dorsal axons (open arrowhead) but no distinct abnormalities in the main axonal trajectories are observed. **C**: 15 μM nicotine exposure (n = 7 fish) causes pathfinding errors when axons reach the lateral periphery of the fish (open arrow). The axons do not properly loop dorsally and they exhibit extensive branching. **D**,**E**: 30 μM nicotine exposure (n = 6 fish) produces various axonal pathfinding errors including failure to project dorsal axons in some segments (D, filled arrow). Pathfinding abnormalities associated with axons extending out along the dorsolateral path and abnormal branching at the ventral root (E, open arrowheads) are also evident. Scale bars = 20 μm.

With the anatomical characterization of the *Tg*(*isl1:GFP*) zebrafish line complete, we examined the long-term consequences of embryonic nicotine exposure on motoneuron development. At 72 hpf, secondary motoneuron axonal trajectories in control (unexposed) larval zebrafish followed their stereotypical paths, extending both medially (“check”-like) and laterally (“loop”-like) into the dorsal myotome, (Fig. 7A). In larval zebrafish (72 hpf) exposed to 5 μM nicotine, the overall pattern of axonal trajectories was not largely affected as the “loop”-like trajectory extended and projected properly into the periphery (Fig. 7B). In some cases, the presence of duplicated “check”-like axonal trajectories exiting the same ventral root was evident (Fig. 7B, open arrowhead). Abnormal trajectories were observed in ≈55% of the total segments analyzed (17 out of 29 segments) in five larval fish (Table 1). A 15-μM nicotine exposure had a more robust effect on axonal trajectories (≈31% of segments analyzed were normal; Table 1) in that some dorsally projecting axons failed to extend completely into the periphery (Fig. 7C, open arrow). Also, accumulation of GFP at the ventral root was evident, indicating axonal stalling at the exit of the root where the axons make their appropriate turns into the periphery. Similar phenotypes were evident in zebrafish embryos exposed to 30 μM nicotine (Fig. 7D,E). In agreement with our previous work (Svoboda et al.,^2002^), some dorsally projecting axons (“check”-like nerve) completely failed to extend into the periphery by 72 hpf (Fig. 7D, filled arrow). Also, in some segments the “loop”-like nerve bundle failed to loop and project along the dorsolateral extent of the fish (Fig. 7E, open arrowheads). GFP accumulation at the exit of the root into the periphery was evident, similar to the 15-μM nicotine exposure. Following a 30-μM nicotine exposure, ≈26% of the segments analyzed had normal axonal trajectories, with the remaining segments exhibiting a wide range of abnormal axonal phenotypes (41 out of 51 segments analyzed were abnormal; Table 1).

**TABLE 1:** Secondary Motoneuron Axon Examination in *isl1* Zebrafish Showing Number of Segments Possessing Their Typical Axonal Morphology

	Fish (n)	Normal† segments	Total segments	Segments with normal† axons (%)
**3 dpf**				
Control	15	79	91	88.93 ± 5.15
5 μM Nicotine	5	12	29	45.83 ± 8.94*
15 μM Nicotine	7	12	36	31.11 ± 5.44*
30 μM Nicotine	7	10	51	26.51 ± 8.20*
**17 dpf**				
Control	5	42	51	84.37 ± 4.41
15 μM Nicotine	5	31	70	47.37 ± 7.54*
30 μM Nicotine	6	19	53	39.32 ± 7.62*

† Axonal trajectories were scored as normal if they exhibited the stereotypical “check”- and “loop”-like trajectories extending either within the dorsomedial or dorsolateral myotome, respectively. Axonal trajectories that would exit spinal cord in between segments, exhibit duplicated “check”-like trajectories and/or stalling, fail to project to the dorsal myotome either medially and/or laterally were scored as not normal. All results are presented as mean ± SE. Asterisks (*) denote significance with a *P*-value < 0.05.

**Figure 8:**
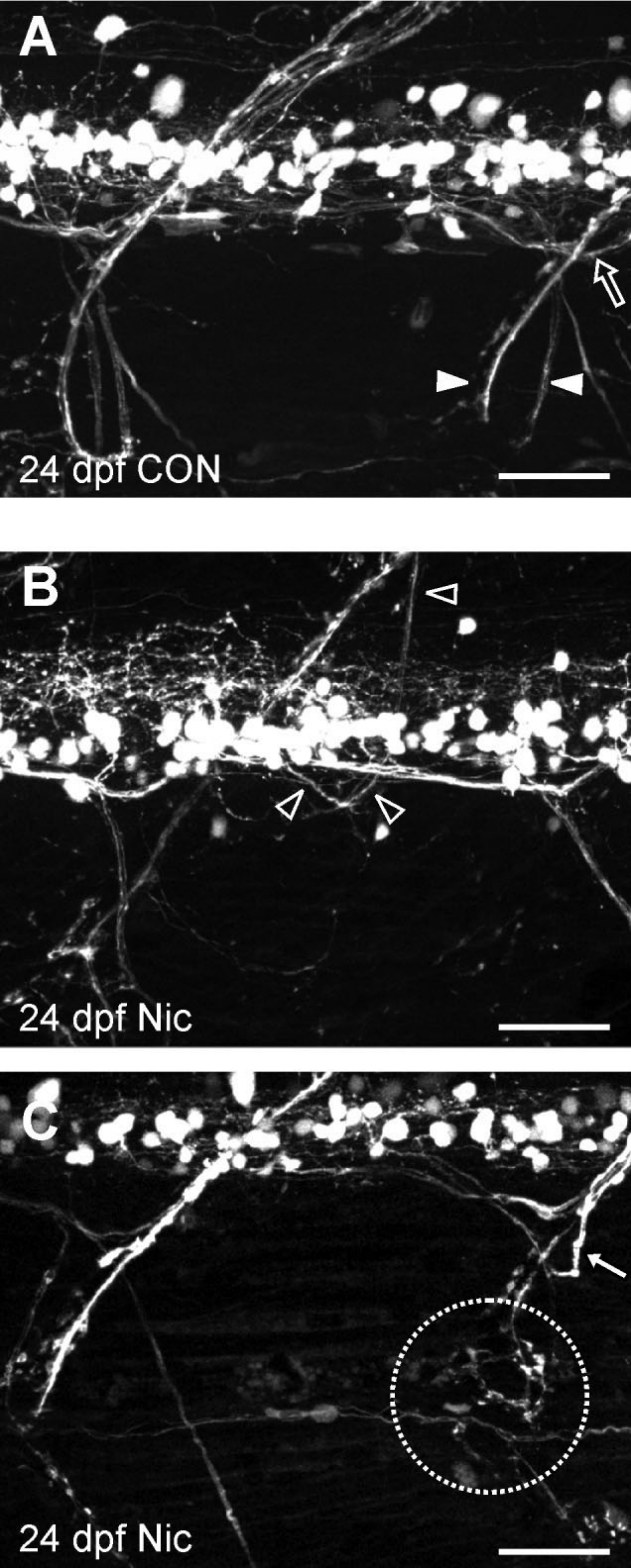
Nicotine-induced abnormalities revealed 3 weeks postexposure in fixed tissue. *Isl1* zebrafish embryos exposed to nicotine were raised to 24 dpf for image examination. **A**: Stage-matched controls (n = 4 fish), exhibit the characteristic trajectories with the “loop”-like (arrowheads) and the “check”-like patterns (open arrow). **B**,**C**: Nicotine exposure (15–30 μM; n = 5 fish) results in abnormal motoneuron trajectories with extra axons exiting spinal cord in between segments (open arrowheads in B). In some cases, duplicated “check”-like trajectories were present (white arrow in C). Also, abnormalities were present at the most lateral region of the fish where the “loop”-like nerve diverges into two distinct axonal trajectories to extend either dorsolaterally or ventrolaterally (circle in C). This disorganization at the most lateral periphery was one of the most frequently encountered nicotine-induced phenotypes. Scale bars = 40 μm.

Axonal pathfinding errors were still evident when analyzed at 24 dpf (3 weeks following nicotine exposure) (Fig. 8). Nicotine-exposed embryos exhibited duplications of the “check”-like nerve in between mid-segmental roots (Fig. 8B, open arrowheads) and also made inappropriate turns into the periphery (Fig. 8C, filled arrow). Abnormalities in the axonal trajectories at the most lateral periphery of the fish were very distinct (Fig. 8C, circle) when compared with their stage-matched controls (Fig. 8A). It appeared that the axons comprising the “loop”-like nerve failed to take their appropriate path and appeared highly disorganized.

**Figure 9:**
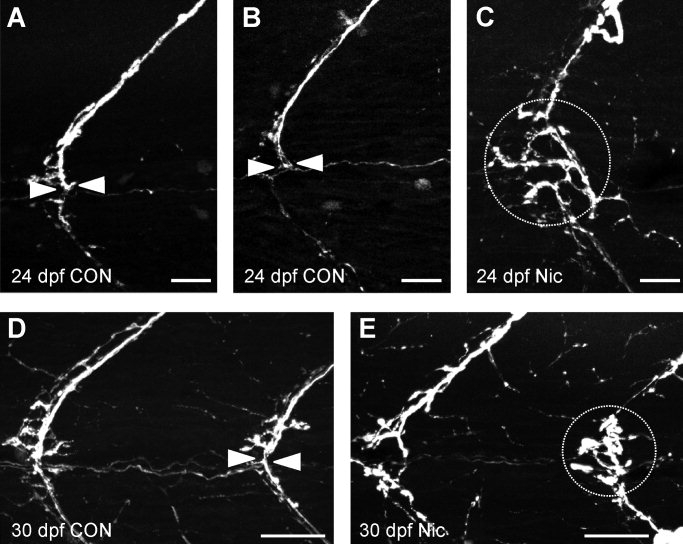
Nicotine-induced abnormalities at the lateral region of juvenile zebrafish. *Isl1* zebrafish embryos exposed to nicotine were raised to 24 dpf and 30 dpf. Images shown are from a series of stacked images focusing on the most lateral motoneuronal trajectories. **A**–**C**: Images of the most lateral motoneuron axonal trajectories in 24-dpf control (A,B, n = 4 fish) and nicotine-exposed (C, n = 5 fish) *isl1* zebrafish. Arrowheads in A and B indicate the distinct choice point at the midline where these axonal trajectories diverge to follow their appropriate paths, dorsally and ventrally, along the segmental boundaries. Embryonic exposure to 30 μM nicotine causes axonal abnormalities at this lateral most region of 24-dpf *isl1* zebrafish. The distinct choice point at the midline is not evident or clear in nicotine-exposed zebrafish where no center point can be distinguished. Rather a highly disorganized pattern is observed which is denoted by the white circle. **D**: Image of a 30-dpf unexposed control *isl1* (n = 4 fish) showing the lateral axonal trajectories with a distinct choice point at the midline indicated by arrowheads. **E**: 30 dpf nicotine-exposed *isl1* zebrafish (n = 6 fish) exhibit severe disorganization at this most lateral region of the fish as seen at 24 dpf. This distinct point is not evident in nicotine-exposed zebrafish and is denoted by the white circle to emphasize this abnormal phenotype observed. Scale bars = 40 μm.

The abnormalities at the most lateral periphery were further examined (Fig. 9). At these later ages the “loop”-like motoneuron axons extend to the lateral most side of the fish where they diverge into two distinct trajectories. One population of axons takes a dorsal path and the other population of axons extends ventrally, resembling a V-shaped pattern (Fig. 9A,B,D). These axons failed to project to this most lateral region of the fish in the stereotypical manner as observed in stage-matched controls. Axonal disorganization in the nicotine-exposed fish was also evident as the “loop”-like axons failed to properly reach the lateral region at the choice point (compare Fig. 9A,B,D arrowheads at the center point and Fig. 9C,E circles) before extending to their appropriate targets (dorsal or ventral) (Fig. 9C,E).

### Nicotine-induced axonal pathfinding errors in *isl1* adult zebrafish

Examination in fixed tissue indicated that embryonic nicotine exposure caused pathfinding errors of secondary motoneurons axons. To eliminate the possibility that that some of the phenotypes observed were fixation artifacts we took advantage of fluorescent stereomicroscopy to image living juvenile zebrafish. This would allow us to identify and track such abnormalities in the same fish as they transitioned into adulthood and determine if these phenotypes were permanent in nature.

**Figure 10:**
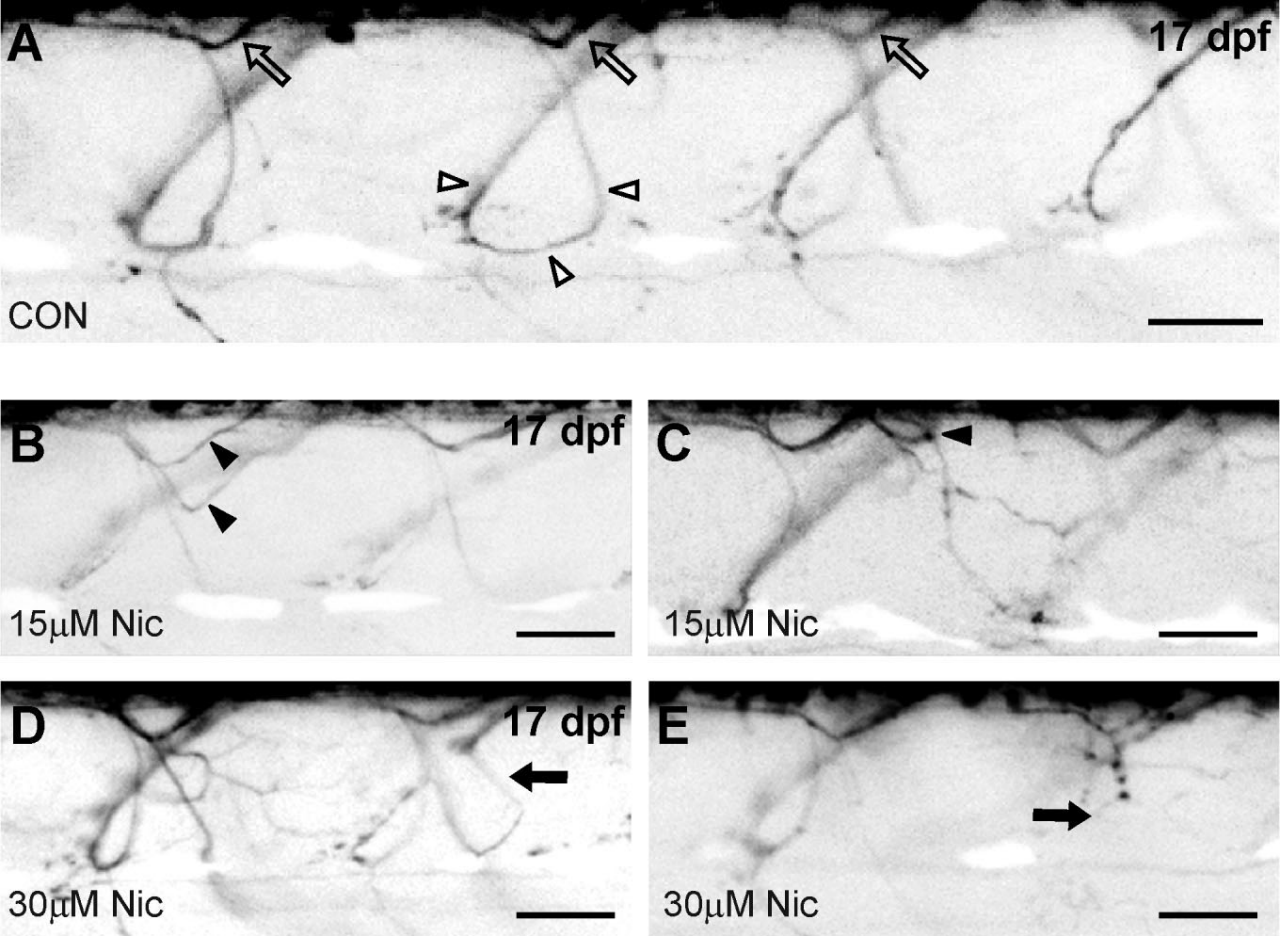
In vivo live imaging in *isl1* zebrafish at 17 dpf reveals nicotine-induced secondary motoneuron axon pathfinding errors. **A**: Representative image of an unexposed control (n = 5) shows the distinct motoneuron axons that project ventrally and then turn laterally (“loop”-like) (open arrowheads) to project into the dorsal musculature. The open arrows point to the “check”-like trajectories that project dorsally and innervate medially located musculature in every segment. **B**,**C**: Photomicrographs of *isl1* zebrafish exposed to 15 μM nicotine (n = 5) show a duplicated axonal trajectory (B, filled arrowheads) and extra axons that exit ventral spinal cord in between segments (C, filled arrowhead). **D**,**E**: *Isl1* zebrafish exposed to 30 μM nicotine (n = 6) exhibit varying abnormal trajectory phenotypes including extra branching medially between segments (D, filled arrow) and stalling of axons extending into the periphery (E, filled arrow). Scale bars = 40 μm.

Live imaging in *isl1* fish 2 weeks following nicotine exposure (17 dpf) revealed the “loop”-like bundles of axons (Fig. 10A, open arrowheads) and the “check”-like trajectories (Fig. 10A, open arrows) in each segment of control zebrafish. *Isl1* zebrafish exposed to 15 μM nicotine often exhibited duplication of the “check”-like nerve (Fig. 10B, arrowheads) and in some instances the “loop”-like axonal trajectories would exit ventral spinal cord at sites other than the common mid-segmental root, often crossing into adjacent segments (Fig. 10C, arrowhead). At the 15 μM nicotine concentration, ≈47% of the segments analyzed exhibited normal axonal trajectories within the myotome (Table 1). Embryonic exposure to 30 μM nicotine produced abnormal axonal targeting (Fig. 10D, filled arrow) and stalling (Fig. 10E, filled arrow). Abnormal axonal phenotypes were observed in 34 out of 53 segments analyzed (≈61%; Table 1).

**Figure 11:**
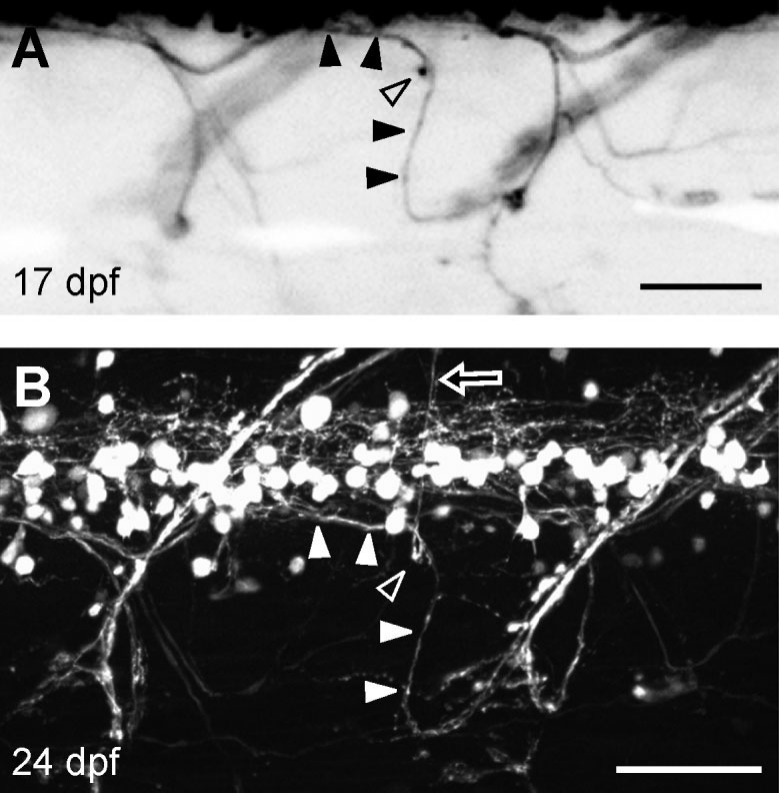
The nicotine-induced abnormalities detected with in vivo live imaging are confirmed in fixed tissue. **A**: Photomicrograph of a 17-dpf *isl1* zebrafish obtained during a live imaging session using fluorescent stereomicroscopy. A duplicated axonal trajectory appears to exit ventral spinal cord via an inter-segmental root and extends to the horizontal myoseptum where the “loop”-like axons take either a dorsolateral or ventrolateral path (filled arrowheads). The same fish was raised until 24 dpf and subsequently analyzed using fluorescent widefield microscopy to obtain z-stacks of images. **B**: Projected image at 24 dpf in the same segments corresponding to the ones observed during live imaging at 17 dpf (A) reveals abnormalities in axonal trajectories (filled arrowheads). A varicosity-like GFP signal (open arrowhead) was evident during live imaging in 17 dpf fish and most likely this GFP signal represents the turning point of an abnormal trajectory which appears to take a sharp dorsal turn within the myotome (open arrow). Scale bars = 40 μm.

We validated the live imaging technique by examining motoneuron anatomy in fixed tissue (24 dpf) from the same fish 1 week after the initial in vivo live imaging (17 dpf). This comparative analysis in the same fish using two microscopy techniques indicated that the phenotypes detected in living fish at 17 dpf (Fig. 11A) still persisted 1 week later, when analyzed in fixed tissue (Fig. 11B). We optimized the live imaging technique so that we could reexamine the same fish over a period of weeks and determine whether the nicotine-induced abnormalities persisted into adulthood. Since we already established that nicotine-induced axonal pathfinding errors persisted from 17 dpf (2 weeks postexposure) to 24 dpf (3 weeks postexposure) (Fig. 11), we hypothesized that the changes were likely permanent. Thus, we aimed to track these changes further into adulthood.

**Figure 12:**
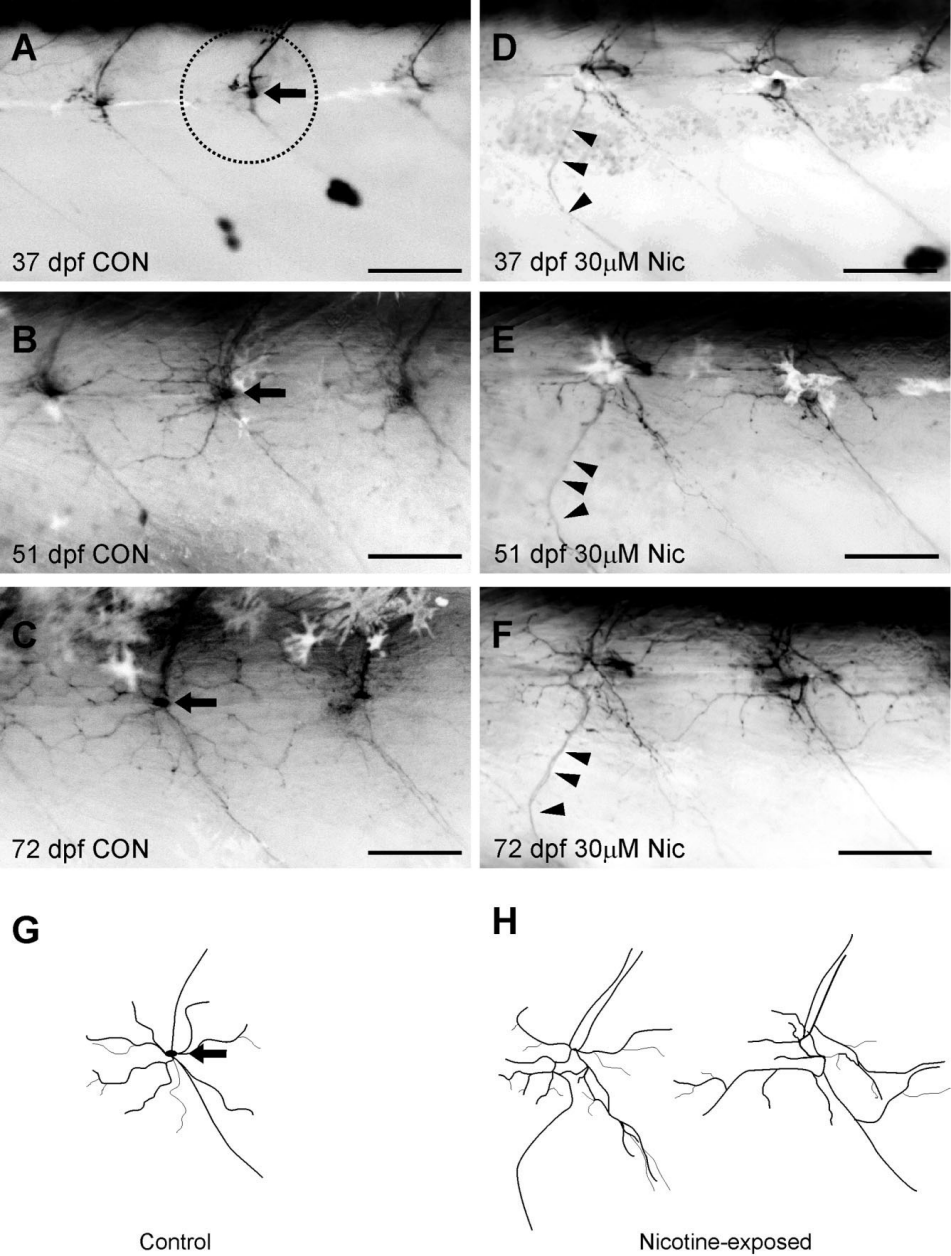
Tracking embryonic nicotine-induced motoneuron axonal changes in adult zebrafish using live imaging in vivo. *Isl1* zebrafish embryos embryonically exposed to 30 μM nicotine were raised into adulthood and monitored for anatomical changes during motoneuron development. Nicotine-exposed (n = 9 fish) and stage-matched controls (n = 5 fish) zebrafish were imaged weekly starting at 37 dpf for 7 weeks (86 dpf). **A**–**C**: Representative photomicrographs of stage-matched controls at 37, 51, and 72 dpf, respectively. **D**–**F**: Nicotine-exposed fish shown at 37, 51, and 72 dpf, respectively. **G**,**H**: Cartoon depicts normal patterns of axonal trajectories in unexposed zebrafish and two examples of nicotine-induced abnormal phenotypes. During these later developmental stages, the unexposed controls have a distinct pattern of dorsally and ventrally projecting axons located at the most lateral part of the fish. This region (black circle in A) corresponds to the region denoted by white arrowheads shown in Figure 9A,B. A varicosity-like GFP signal (GFP “center spot”) (A–C, filled arrows) is evident with additional axons branching from the “center spot” out between segments to where the “loop”-like trajectories extend laterally (perpendicular and out the plane of the page) from the medial myotome. In nicotine-exposed fish, these motoneuron axons have a disorganized patterning associated with the center point at the most lateral periphery. Axons also exhibit crossing over into adjacent segments (D–F, arrowheads) when compared to their stage-matched controls. Cartoon in D is not shown to scale. Scale bars = 100 μm.

*Isl1* zebrafish and their stage-matched controls were exposed to nicotine as embryos and raised until 37 dpf (≈5 weeks postexposure). Starting at this age, live imaging was performed every week for a total of 7 weeks (37–86 dpf) (Fig. 12 and data not shown). At these later stages of development we focused our live imaging analysis to the most lateral region of the fish, primarily because the GFP positive axons located medially deep in the trunk musculature became obscured as the zebrafish got thicker (see Suppl. Fig. 2). Moreover, we already demonstrated nicotine-induced abnormal phenotypes in the axonal trajectories originating from the “loop”-like nerve at the lateral most part of the myotome (refer to Figs. 8, 9). Abnormalities in these most lateral axonal trajectories were also detected at 37 dpf (Fig. 12D). Anatomical analysis revealed a great degree of disorganization at the lateral extent of this axonal trajectory when compared to the analogous trajectory in stage-matched controls (Fig. 12A). Live imaging in unexposed zebrafish identified a varicosity-like GFP signal (GFP “center spot”) (Fig. 12A–C, arrow) where the “loop”-like axonal trajectories extend laterally (perpendicular and out of the plane of the page) from the medial myotome. The GFP accumulation at the “center spot” was always present as the zebrafish transitioned into early adulthood. Secondary and tertiary branches extended radially from this center point to innervate adjacent muscle fibers (Fig. 12G). This axonal pattern was severely disrupted in nicotine-exposed zebrafish with no distinguishable GFP “center spot” (Fig. 12H).

Unexposed fish exhibited axonal branching extending from the GFP “center spot” out into the lateral myotome, but this was confined within the segmental boundaries. Axons in nicotine-exposed zebrafish often exhibited crossing into adjacent segments (Fig. 12D–F, arrowheads) which was never observed in any of the unexposed controls.

## DISCUSSION

The presence of molecular machinery for synthesizing acetylcholine (ACh) and responding to it during critical periods of embryogenesis suggests that cholinergic stimulation via activation of nAChRs is very important early in development. Coexpression of multiple subunit transcripts with functional nAChRs was detected in premigratory neural crest cells (Howard et al.,^1995^) and in autonomic neurons early in development (Devay et al.,^1994^). There is evidence that nicotine exposure during vulnerable developmental stages can also lead to programmed cell death (apoptosis) (Berger et al.,^1998^; Roy et al.,^1998^) and inhibit DNA synthesis in the brain (Slotkin,^1998^).

We have been studying the consequences of nicotine exposure on vertebrate development using the zebrafish model system. Zebrafish embryos develop entirely outside the mother, which allows accessibility at all stages of development. Thus, we have great control over nicotine administration into the embryo and, if needed, the nicotine uptake can be systematically quantified. Also, the locomotor behaviors of escape and swimming have been well characterized in teleost fish including zebrafish (Downes and Granato,^2006^; Fetcho,^2007^; Fetcho et al.,^2008^). This makes the zebrafish model well suited to pursue research objectives where nicotine-induced perturbations in anatomy and physiology can be monitored from embryonic stages of development into adulthood with the goal of linking those perturbations to long-term changes in behavior.

Only a few researchers have characterized motoneuron axonal trajectories and innervation patterns in adult zebrafish (Thorsen and Hale,^2007^). In that work, the innervation pattern of pectoral fin muscles by motoneurons was analyzed. Ultimately, our research objective is to examine the impact of nicotine exposure not only at the anatomical level but also at the behavioral and physiological levels. Consequently, we have focused on characterizing the anatomy of zebrafish spinal neurons associated with locomotion.

We took advantage of fluorescent microscopy techniques combined with genetically engineered reporter zebrafish (*isl1*) to visualize nicotine-induced alterations in neuronal structures in juvenile and adult zebrafish. We first characterized spinal motoneuron anatomy in larval and juvenile *isl1* zebrafish. This was performed with the aid of widefield fluorescent microscopy in *isl1* zebrafish (3–30 dpf). We then established an anatomical model that described four spatially distinct axonal trajectories distributed within the trunk musculature of *isl1* zebrafish. These axonal trajectories take their appropriate paths in the periphery in a highly organized and stereotypical manner from early (embryonic and larval) to later (juvenile and adult) stages of development.

We found that nicotine exposure resulted in permanent changes in axonal pathfinding examined in the *isl1* GFP reporter line. These pathfinding errors were first detected in larval zebrafish at the end of the nicotine exposure window (72 hpf). These nicotine-induced abnormalities in axonal targeting persisted into juvenile and almost adult stages in zebrafish. The live imaging technique established in this study allowed us to detect nicotine-induced abnormalities and also to monitor and track these changes over time in the same fish as late as 86 days of age. Although applying these techniques in zebrafish is not necessarily unique (Distel et al.,^2006^), we believe that this is the first time that a nicotine-induced alteration in neuronal anatomy caused by embryonic exposure has been monitored throughout vertebrate development and imaged in a living model system.

The mechanism underlying nicotine's ability to cause permanent changes in axonal pathfinding was not a primary focus of this study. However, there are two likely scenarios that may account for the long-term nature of the nicotine-induced changes. One possible mechanism may be related to changes in muscle development caused by nicotine exposure. In the zebrafish mutant known as *twister*, a gain-of-function mutation in the chrna1 gene encoding the α-subunit of the muscle-specific AChR, causes the receptor to have longer open times upon binding acetylcholine. This increased muscular activity causes muscle fiber degeneration and motoneuron axonal pathfinding errors (Lefebvre et al.,^2004^). In this context, exposure to nicotine can also directly overactivate skeletal muscle nicotinic acetylcholine receptors. Nicotine exposures (15–30 μM) during the same exposure window used in this study can also lead to muscle degeneration and atrophy (Welsh et al.,^2008^). The nicotine-induced motoneuron and muscle abnormalities occur in parallel with the possibility that each phenotype exacerbates the other. In *sofa potato* (*sop*) paralytic mutants lacking functional muscle AChRs, motoneuron axonal pathfinding is normal even in the absence of muscle activity (Ono et al.,^2001^; Welsh et al.,^2008^). However, when *sop* mutants are exposed to nicotine (15–30 μM) they exhibit motoneuron pathfinding errors without any concurrent muscle degeneration (Welsh et al.,^2008^). This suggests that muscle overactivity and degeneration is not the exclusive source contributing to the axonal pathfinding errors and that nicotine can directly activate neuronal substrates, possibly motoneurons. Consistent with this, low concentrations of nicotine (5 μM) can induce motoneuron axonal pathfinding errors (this study) with no effect on muscle morphology (unpubl. obs.). This further indicates that nicotine can act directly on the CNS to alter motoneuron axon pathfinding.

The long-term abnormalities in motoneuron axonal pathfinding detected in juvenile and adult zebrafish are likely caused by direct action of nicotine on motoneurons and concurrent overactivation of muscle AChR leading to degeneration. However, if the nicotine-induced degeneration in the muscle is never restored, then this could also contribute to new motoneuron axon pathfinding errors. This would be true of motoneuron axons that begin targeting after the nicotine has been withdrawn. If those axons are destined to target muscle that has now become atrophic due to the nicotine, they would need to be redirected to healthy muscle.

Since nicotine can directly act on the CNS, nicotine-induced fluctuations in calcium homeostasis may also contribute to axonal pathfinding errors. It is known that the nAChR agonists nicotine and acetylcholine can regulate neurite outgrowth in ciliary ganglion neurons (Pugh and Berg,^1994^). In isolated *Xenopus* spinal neurons, acetylcholine-induced turning of nerve growth cones is calcium-dependent (Zheng et al.,^1994^). Typically, acetylcholine-induced calcium influx is required for extension and turning of growth cones, but a sudden increase in intracellular calcium induces retraction of neurites (Zheng et al.,^1994^). Also, the frequency of calcium transients in growth cones is inversely proportional to their rate of migration and extension (Gomez and Spitzer,^1999^). In zebrafish embryos exposed to nicotine the presence of nicotine may overactivate nAChRs during a critical developmental window when motoneuron growth cones are migrating and extending in the periphery. This potentially can disrupt the normal calcium homeostasis leading to abnormal targeting into the periphery due to axon stalling and/or retraction. Consistent with this, we have identified nAChR subunits on motoneuron axons in developing zebrafish (Welsh et al.,^2008^). This may provide the substrate at the level of the CNS that nicotine acts on to influence axonal pathfinding.

Once the motoneuronal trajectories take inappropriate paths into the periphery with no direction or guidance at the time that is most critical, they are then likely to be permanently altered, as demonstrated by this study. We are currently performing experiments to unravel the mechanism(s) underlying the nicotine-induced long-term abnormalities in zebrafish motoneuron axonal trajectories.

## CONCLUSIONS

In this study we set out to determine whether nicotine-induced abnormalities in axonal anatomy were permanent and lasted into adulthood. We established a live imaging technique whereby we could identify and subsequently track nicotine-induced motoneuronal changes in the same animal throughout development. Our results clearly show that nicotine exposure during a critical window of development can lead to axonal pathfinding errors that are likely permanent in nature. When motoneuron axons exhibit abnormal trajectories during early stages of development, the abnormalities will most likely last into adulthood. Even though nicotine can directly act on motoneurons to induce these errors, we still need to consider that muscle degeneration may also contribute to new abnormal motoneuron phenotypes during later stages of development.

## References

[b1] Beattie CE, Hatta K, Halpern ME, Liu H, Eisen JS, Kimmel CB (1997). Temporal separation in the specification of primary and secondary motoneurons in zebrafish. Dev Biol.

[b2] Berger F, Gage FH, Vijayaraghavan S (1998). Nicotinic receptor-induced apoptotic cell death of hippocampal progenitor cells. J Neurosci.

[b3] Bhatt DH, Otto SJ, Depoister B, Fetcho JR (2004). Cyclic AMP-induced repair of zebrafish spinal circuits. Science.

[b4] Burns FR, von Kannen S, Guy L, Raper JA, Kamholz J, Chang S (1991). DM-GRASP, a novel immunoglobulin superfamily axonal surface protein that supports neurite extension. Neuron.

[b5] Chen Q, Sealock R, Peng HB (1990). A protein homologous to the Torpedo postsynaptic 58K protein is present at the myotendinous junction. J Cell Biol.

[b6] Crow MT, Stockdale FE (1986). Myosin expression and specialization among the earliest muscle fibers of the developing avian limb. Dev Biol.

[b7] Devay P, Qu X, Role L (1994). Regulation of nAChR subunit gene expression relative to the development of pre- and postsynaptic projections of embryonic chick sympathetic neurons. Dev Biol.

[b8] Devoto SH, Melançon E, Eisen JS, Westerfield M (1996). Identification of separate slow and fast muscle precursor cells in vivo, prior to somite formation. Development.

[b9] DiFranza JR, Lew RA (1995). Effect of maternal cigarette smoking on pregnancy complications and sudden infant death syndrome. J Fam Pract.

[b10] Distel M, Babaryka A, Köster RW (2006). Multicolor in vivo time-lapse imaging at cellular resolution by stereomicroscopy. Dev Dyn.

[b11] Downes GB, Granato M (2006). Supraspinal input is dispensable to generate glycine-mediated locomotive behaviors in the zebrafish embryo. J Neurobiol.

[b12] Fashena D, Westerfield M (1999). Secondary motoneuron axons localize DM-GRASP on their fasciculated segments. J Comp Neurol.

[b13] Fetcho JR (1986). The organization of the motoneurons innervating the axial musculature of vertebrates. I. Goldfish (Carassius auratus) and mudpuppies (Necturus maculosus). J Comp Neurol.

[b14] Fetcho JR (2007). The utility of zebrafish for studies of the comparative biology of motor systems. J Exp Zool B Mol Dev Evol.

[b15] Fetcho JR, Higashijima S, McLean DL (2008). Zebrafish and motor control over the last decade. Brain Res Rev.

[b16] Gomez TM, Spitzer NC (1999). In vivo regulation of axon extension and pathfinding by growth-cone calcium transients. Nature.

[b17] Hale ME, Ritter DA, Fetcho JR (2001). A confocal study of spinal interneurons in living larval zebrafish. J Comp Neurol.

[b18] Higashijima S, Hotta Y, Okamoto H (2000). Visualization of cranial motor neurons in live transgenic zebrafish expressing green fluorescent protein under the control of the islet-1 promoter/enhancer. J Neurosci.

[b19] Hory-Lee F, Frank E (1995). The nicotinic blocking agents d-tubocurare and alpha-bungarotoxin save motoneurons from naturally occurring death in the absence of neuromuscular blockade. J Neurosci.

[b20] Howard MJ, Gershon MD, Margiotta JF (1995). Expression of nicotinic acetylcholine receptors and subunit mRNA transcripts in cultures of neural crest cells. Dev Biol.

[b21] Kawahara A, Chien CB, Dawid IB (2002). The homeobox gene mbx is involved in eye and tectum development. Dev Biol.

[b22] Kerschensteiner M, Schwab ME, Lichtman JW, Misgeld T (2005). In vivo imaging of axonal degeneration and regeneration in the injured spinal cord. Nat Med.

[b23] Lawson ND, Weinstein BM (2002). In vivo imaging of embryonic vascular development using transgenic zebrafish. Dev Biol.

[b24] Lefebvre JL, Ono F, Puglielli C, Seidner G, Franzini-Armstrong C, Brehm P, Granato M (2004). Increased neuromuscular activity causes axonal defects and muscular degeneration. Development.

[b25] Lewis KE, Eisen JS (2003). From cells to circuits: development of the zebrafish spinal cord. Prog Neurobiol.

[b26] Meng A, Tang H, Ong BA, Farrell MJ, Lin S (1997). Promoter analysis in living zebrafish embryos identifies a cis-acting motif required for neuronal expression of GATA-2. Proc Natl Acad Sci U S A.

[b27] Myers PZ (1985). Spinal motoneurons of the larval zebrafish. J Comp Neurol.

[b28] Myers PZ, Eisen JS, Westerfield M (1986). Development and axonal outgrowth of identified motoneurons in the zebrafish. J Neurosci.

[b29] Ono F, Higashijima S, Shcherbatko A, Fetcho JR, Brehm P (2001). Paralytic zebrafish lacking acetylcholine receptors fail to localize rapsyn clusters to the synapse. J Neurosci.

[b30] Ono F, Mandel G, Brehm P (2004). Acetylcholine receptors direct rapsyn clusters to the neuromuscular synapse in zebrafish. J Neurosci.

[b31] Ott H, Diekmann H, Stuermer CA, Bastmeyer M (2001). Function of Neurolin (DM-GRASP/SC-1) in guidance of motor axons during zebrafish development. Dev Biol.

[b32] Paz R, Barsness B, Martenson T, Tanner D, Allan AM (2007). Behavioral teratogenicity induced by nonforced maternal nicotine consumption. Neuropsychopharmacology.

[b33] Pike SH, Melancon EF, Eisen JS (1992). Pathfinding by zebrafish motoneurons in the absence of normal pioneer axons. Development.

[b34] Pineda RH, Svoboda KR, Wright MA, Taylor AD, Novak AE, Gamse JT, Eisen JS, Ribera AB (2006). Knockdown of Nav1.6a Na+ channels affects zebrafish motoneuron development. Development.

[b35] Pugh PC, Berg DK (1994). Neuronal acetylcholine receptors that bind alpha-bungarotoxin mediate neurite retraction in a calcium-dependent manner. J Neurosci.

[b36] Roy TS, Andrews JE, Seidler FJ, Slotkin TA (1998). Nicotine evokes cell death in embryonic rat brain during neurulation. J Pharmacol Exp Ther.

[b37] Slikker W, Xu ZA, Levin ED, Slotkin TA (2005). Mode of action: disruption of brain cell replication, second messenger, and neurotransmitter systems during development leading to cognitive dysfunction–developmental neurotoxicity of nicotine. Crit Rev Toxicol.

[b38] Slotkin TA (1998). Fetal nicotine or cocaine exposure: which one is worse?. J Pharmacol Exp Ther.

[b39] Slotkin TA, Saleh JL, McCook EC, Seidler FJ (1997). Impaired cardiac function during postnatal hypoxia in rats exposed to nicotine prenatally implications for perinatal morbidity and mortality, and for sudden infant death syndrome. Teratology.

[b40] Svoboda KR, Linares AE, Ribera AB (2001). Activity regulates programmed cell death of zebrafish Rohon-Beard neurons. Development.

[b41] Svoboda KR, Vijayaraghavan S, Tanguay RL (2002). Nicotinic receptors mediate changes in spinal motoneuron development and axonal pathfinding in embryonic zebrafish exposed to nicotine. J Neurosci.

[b42] Thapar A, Fowler T, Rice F, Scourfield J, van den Bree M, Thomas H, Harold G, Hay D (2003). Maternal smoking during pregnancy and attention deficit hyperactivity disorder symptoms in offspring. Am J Psychiatry.

[b43] Thorsen DH, Hale ME (2007). Neural development of the zebrafish (Danio rerio) pectoral fin. J Comp Neurol.

[b44] Welsh L, Tanguay RL, Svoboda KR (2008). Uncoupling nicotine mediated motoneuron axonal path-finding errors from muscle degeneration in zebrafish. Toxicol Appl Pharmacol.

[b45] Westerfield M (1995). The zebrafish book.

[b46] Westerfield M, McMurray JV, Eisen JS (1986). Identified motoneurons and their innervation of axial muscles in the zebrafish. J Neurosci.

[b47] Yaniv K, Isogai S, Castranova D, Dye L, Hitomi J, Weinstein BM (2006). Live imaging of lymphatic development in the zebrafish. Nat Med.

[b48] Zeller J, Schneider V, Malayaman S, Higashijima S, Okamoto H, Gui J, Lin S, Granato M (2002). Migration of zebrafish spinal motor nerves into the periphery requires multiple myotome-derived cues. Dev Biol.

[b49] Zhang G, Jin L-Q, Sul J-Y, Haydon PG, Selzer ME (2005). Live Imaging of Regenerating Lamprey Spinal Axons. Neurorehabil Neural Repair.

[b50] Zheng JQ, Felder M, Connor JA, Poo MM (1994). Turning of nerve growth cones induced by neurotransmitters. Nature.

