# Supplementary figures and images for: Secondary motoneurons in juvenile and adult zebrafish: Axonal pathfinding errors caused by embryonic nicotine exposure
Source: J Comp Neurol. 2009 Jan 20;512(3):305–22. doi: 10.1002/cne.21903 (PMC2798059; doi:10.1002/cne.21903)

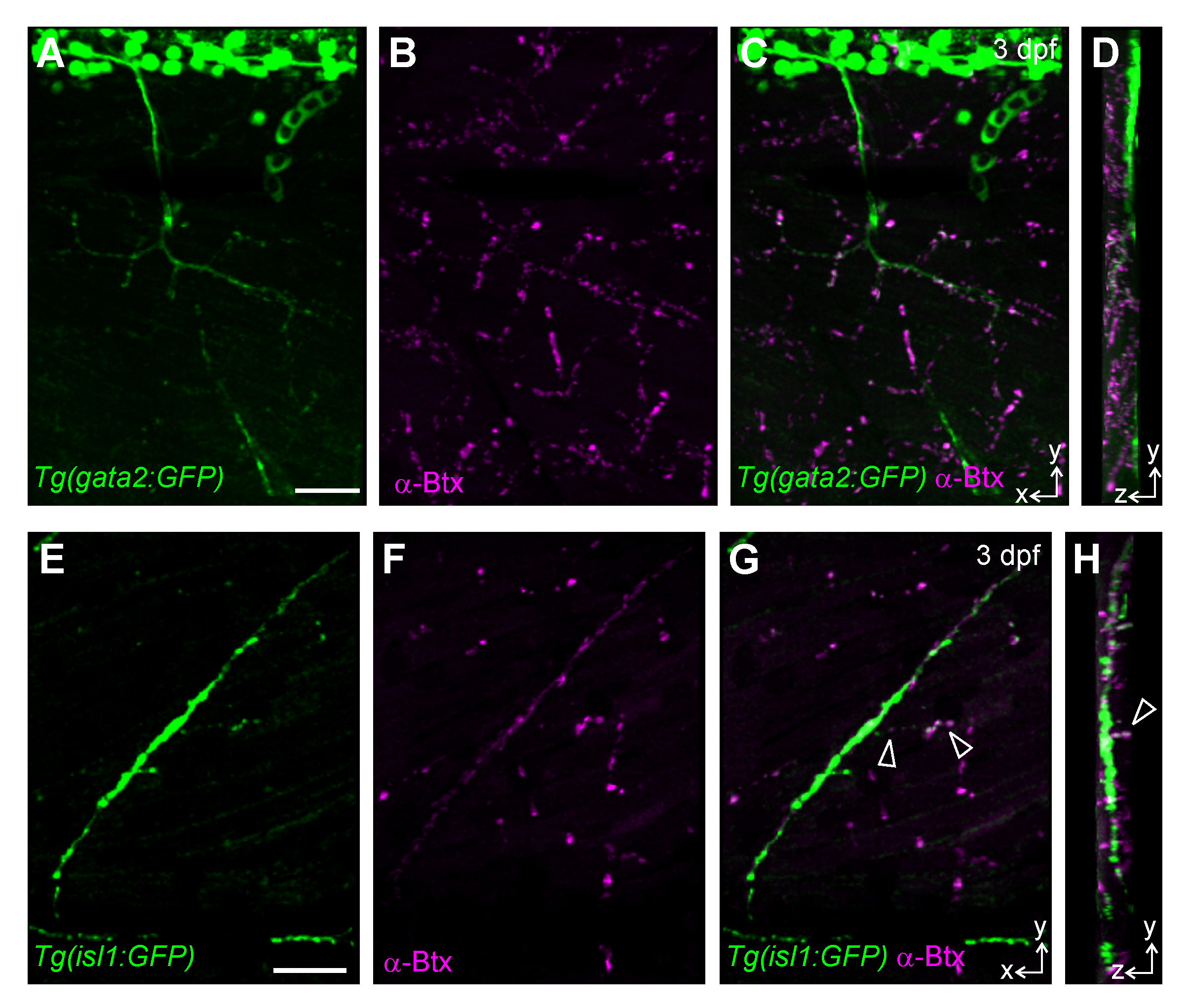

Supplement: Supplementary file 1 [file cne0512-0305-SD1.tif]

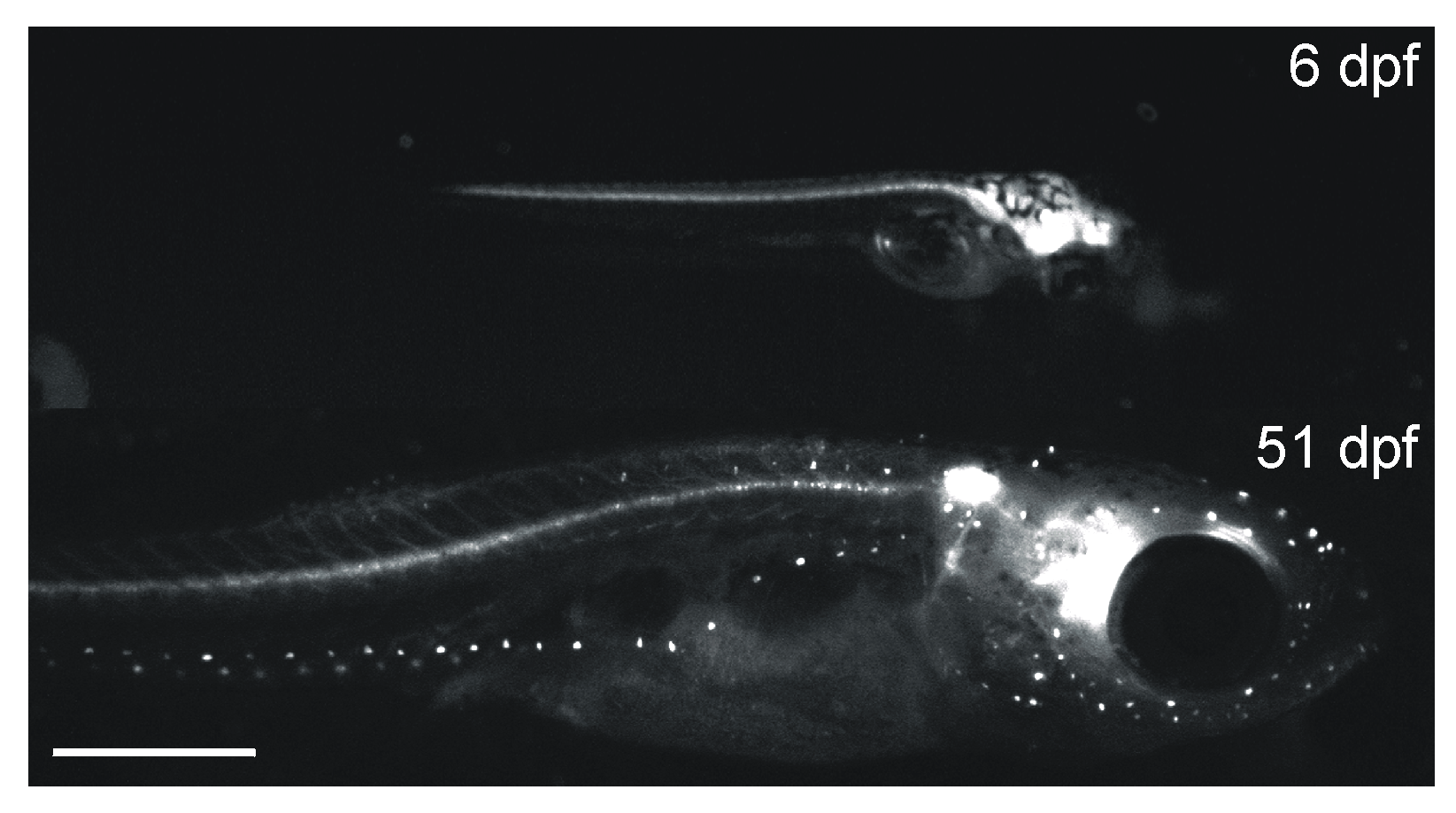

Supplement: Supplementary file 2 [file cne0512-0305-SD2.tif]

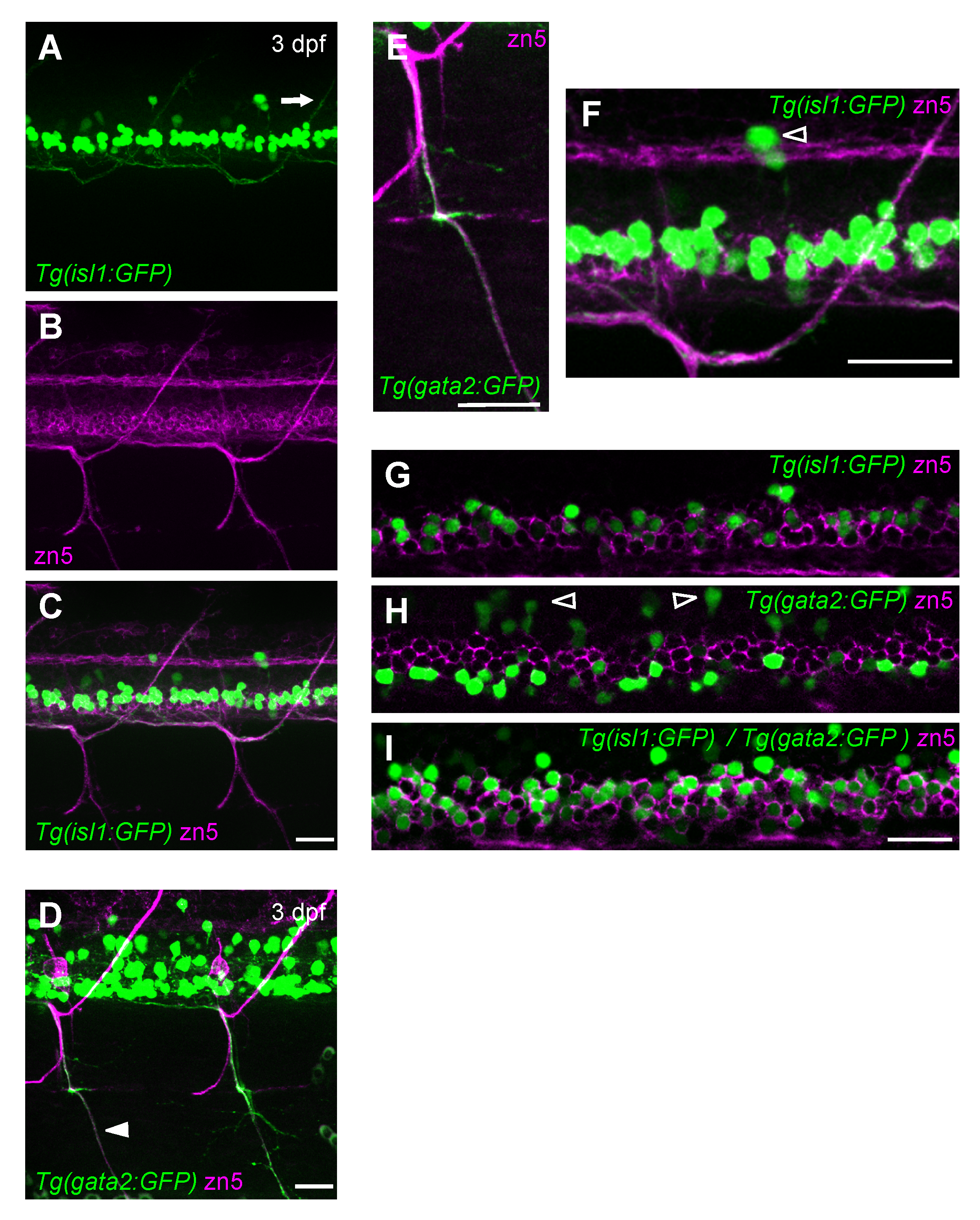

Supplement: Supplementary file 3 [file cne0512-0305-SD3.tif]

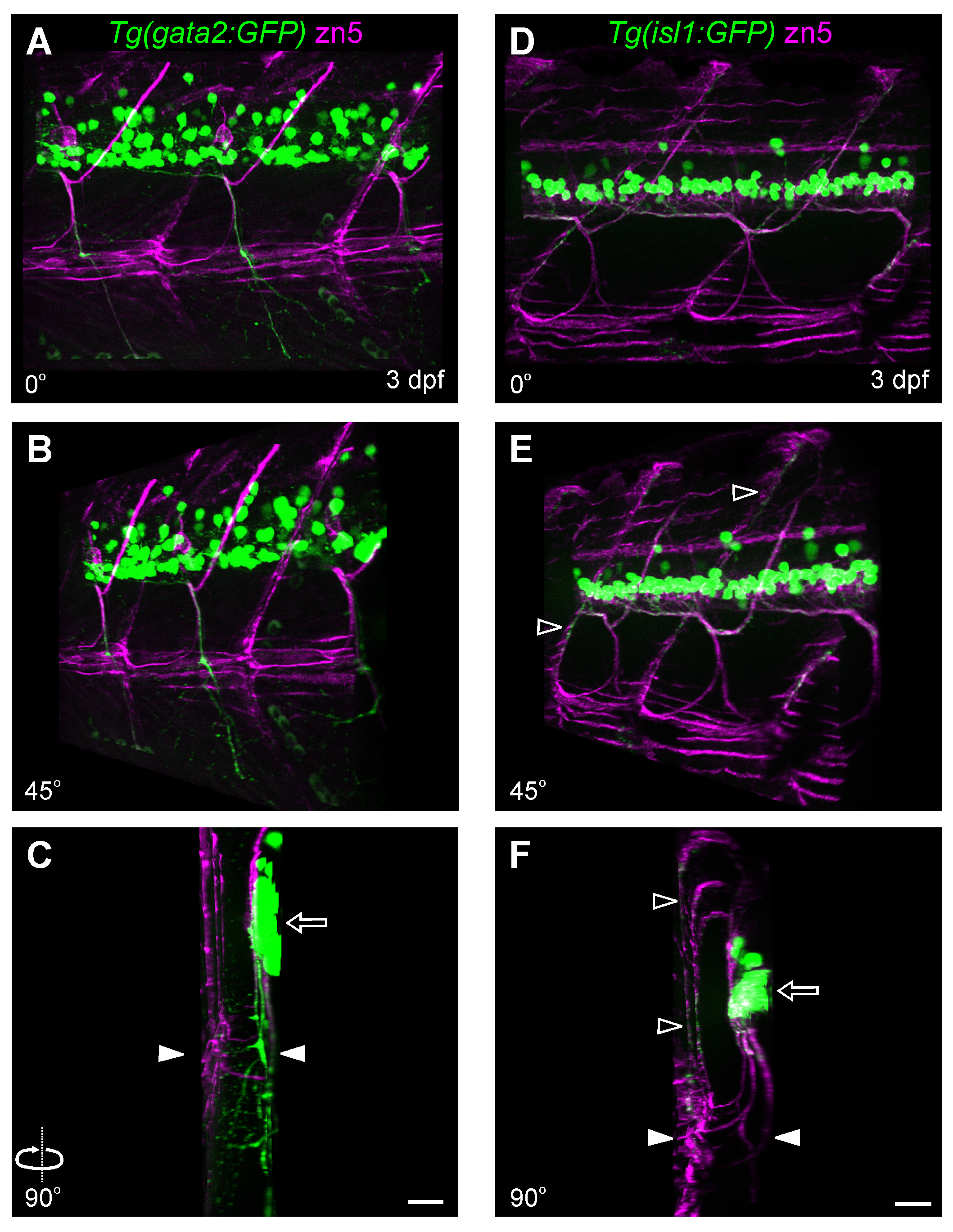

Supplement: Supplementary file 4 [file cne0512-0305-SD4.tif]

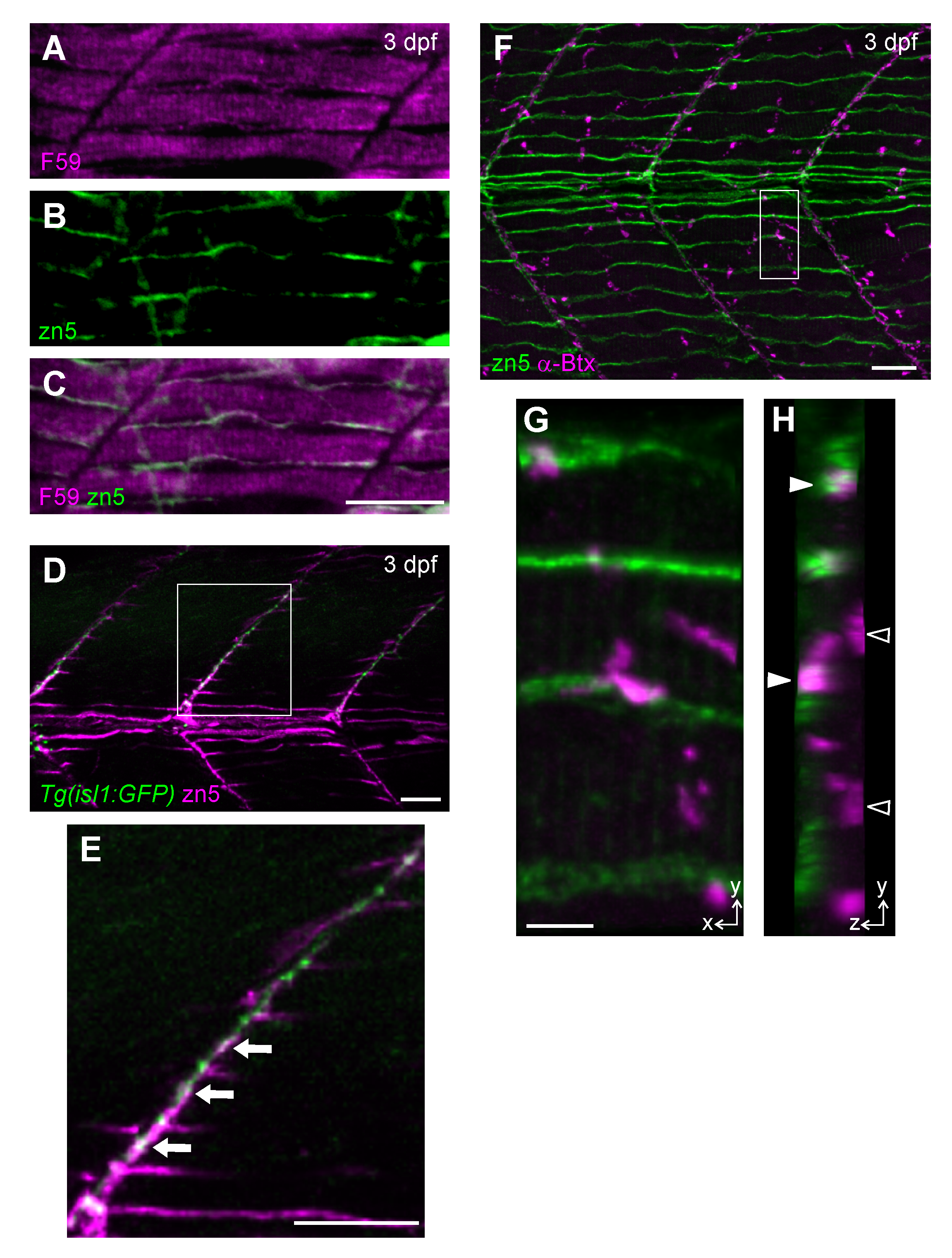

Supplement: Supplementary file 5 [file cne0512-0305-SD5.tif]

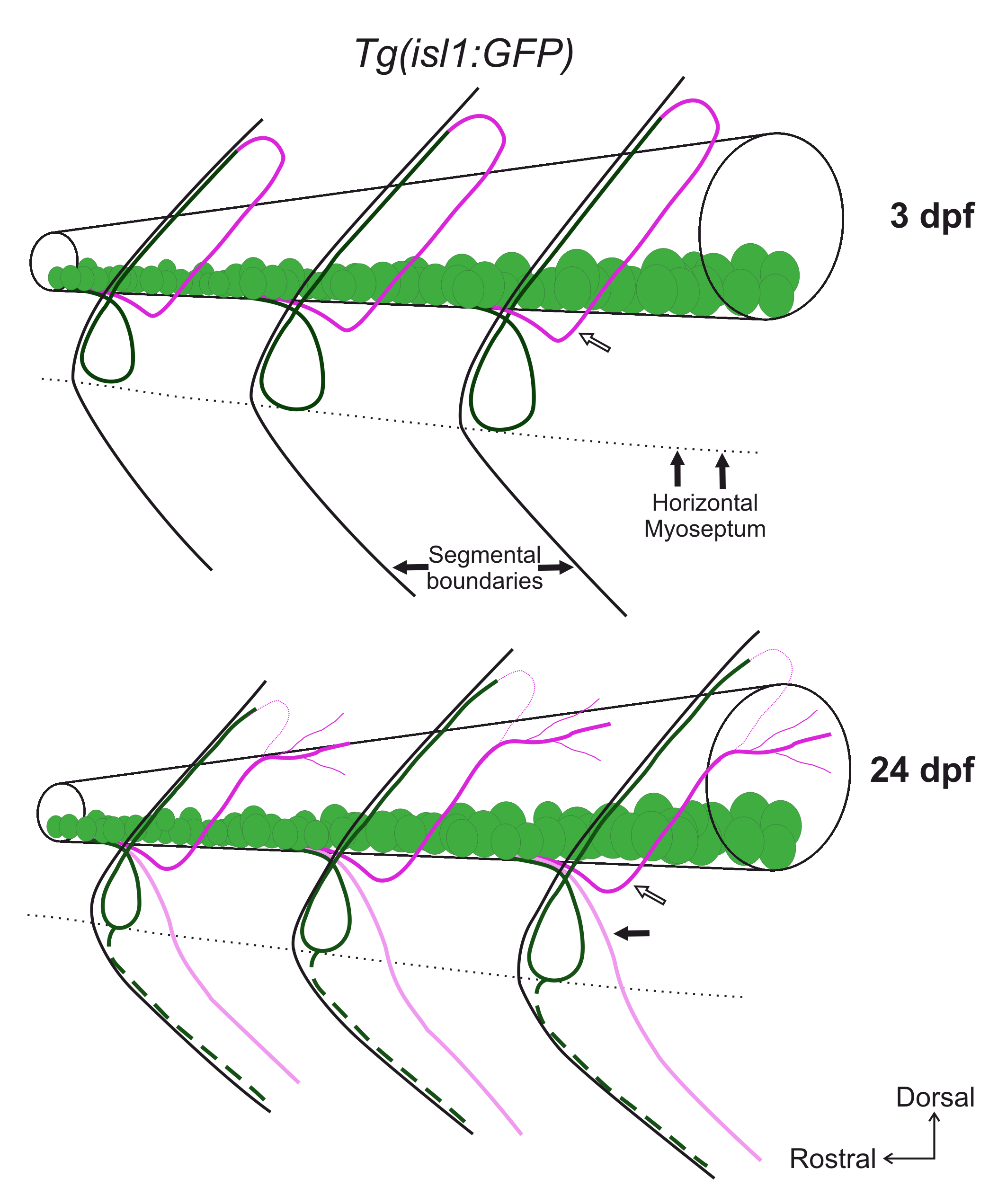

Supplement: Supplementary file 6 [file cne0512-0305-SD6.tif]
